# A synthetic benzoxazine dimer derivative targets c‐Myc to inhibit colorectal cancer progression

**DOI:** 10.1002/1878-0261.70127

**Published:** 2025-10-14

**Authors:** Nicharat Sriratanasak, Bodee Nutho, Worawat Wattanathana, Narumon Phaonakrop, Bunnatut Panasawatwong, Katharina Erlenbach‐Wuensch, Sittiruk Roytrakul, Regine Schneider‐Stock, Pithi Chanvorachote

**Affiliations:** ^1^ Faculty of Pharmaceutical Sciences, Department of Pharmacology and Physiology Chulalongkorn University Bangkok Thailand; ^2^ Faculty of Pharmaceutical Sciences, Center of Excellence in Cancer Cell and Molecular Biology Chulalongkorn University Bangkok Thailand; ^3^ Experimental Tumorpathology University Hospital Erlangen, Friedrich‐Alexander University Erlangen‐Nürnberg Germany; ^4^ Institute of Pathology University Hospital Erlangen, Friedrich‐Alexander University Erlangen‐Nürnberg Germany; ^5^ Faculty of Science, Department of Pharmacology Mahidol University Bangkok Thailand; ^6^ Faculty of Engineering, Department of Materials Engineering Kasetsart University Bangkok Thailand; ^7^ Functional Proteomics Technology Laboratory National Center for Genetic Engineering and Biotechnology, National Science and Technology Development Agency Pathumthani Thailand 12120; ^8^ Comprehensive Cancer Center Erlangen‐EMN (CCC ER‐EMN) Germany; ^9^ Adjunct professor at Mahidol University Bangkok Thailand; ^10^ Sustainable Environment Research Institute Chulalongkorn University Bangkok Thailand

**Keywords:** cell cycle arrest, c‐Myc inhibitor, colorectal cancer, DNA damage, ECD, lead structure, protein destabilization, ubiquitin‐proteasomal degradation

## Abstract

The c‐Myc protein is a well‐known oncoprotein that plays a crucial role in regulating cell growth, proliferation, and differentiation. The overexpression or dysregulation of c‐Myc is commonly associated with tumorigenesis in several cancers, including colorectal cancer (CRC). c‐Myc forms a heterodimer with its partner MAX to activate the expression of various genes. Here, we synthesized a novel c‐Myc‐targeting small molecule, 2,2′‐((cyclohexylazanedyl)bis(methylene))bis(4‐ethylphenol), or ECD, and demonstrate ECD's anticancer activity via interference with the c‐Myc/MAX dimer to promote c‐Myc degradation in CRC cells *in vitro*, *in silico*, and *in vivo*. This study revealed the activity of ECD toward CRC cells as a c‐Myc inhibitor. Computer‐aided analysis revealed that the effect of ECD was mediated through disturbance of the c‐Myc/MAX complex. Moreover, ECD exhibited cytotoxic activity by inducing DNA damage, leading to apoptotic cell death. This DNA damage‐inducing property was also confirmed by whole‐proteome profiling of HT29 cells after ECD treatment. In the chick embryo chorioallantoic membrane (CAM) xenograft assay, we demonstrated a remarkable inhibition of the tumorigenic activity upon ECD exposure. In summary, we identified ECD as a novel potent compound targeting the oncoprotein c‐Myc that may offer new opportunities for CRC treatment.

## Graphical abstract

Benzoxazine dimer derivatives bind to the bHLH‐LZ region of c‐Myc, disrupting c‐Myc/MAX complexes, which are evaluated from SAR analysis. This increases ubiquitination and reduces cellular c‐Myc. Impairing DNA repair mechanisms is shown through proteomic analysis. *In vitro* and *in ovo* studies demonstrate tumorigenesis inhibition through cell cycle arrest and apoptosis.

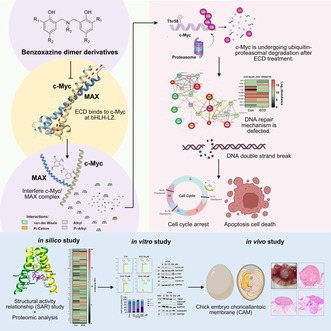

**Abbreviations**
Chk‐1: checkpoint kinase 1
CYLD: ubiquitin carboxyl‐terminal hydrolase CYLD
DAPI: 4',6‐diamidino‐2‐phenylindole
DMSO: dimethyl sulfoxide
DSB: double strand break
ECD: 2; 2'–((cyclohexylazanedyl) bis(methylene)) bis(4‐ethylphenol)
EMD: 2,2'‐((methylazanedyl)bis(methylene)) bis(4‐ethylphenol)
FFPE: formalin‐fixed paraffin‐embedded
H3K9me3: histone 3 lysine 9 trimethylation
MCL1: induced myeloid leukemia cell differentiation protein Mcl‐1
MRE11A: meiotic recombination 11 homolog A
MRNIP: MRN complex‐interacting protein
MYH14: myosin‐14
PARP: poly(ADP‐ribose) polymerase
p‐Chk1: phosphorylated checkpoint kinase 1
PI: propidium iodide
RPA1: replication protein A 70 kDa DNA‐binding subunit
TOP2A: DNA topoisomerase 2‐alpha
USP37: ubiquitin carboxyl‐terminal hydrolase 37
γ‐H2AX: phosphorylated histone 2AX

## 1 Introduction

Colorectal cancer (CRC) is the second most prevalent public health issue worldwide. In 2023, approximately 30% of diagnosed patients were anticipated to die from this disease. CRC is increasingly being diagnosed in younger patients and is most often first detected in advanced stages. Almost 1.5 million patients are newly diagnosed with CRC annually, and almost 50 thousand patients die even though improved diagnostic techniques can detect the disease at an earlier stage [1, 2]. The activation of oncoproteins together with the inactivation of tumor suppressor proteins is the generally accepted mechanism of colorectal tumorigenesis. Currently, a therapeutic option for cancer is targeted therapy because it can extend the overall survival time of CRC patients [3]. It has been reported that combinations of targeted therapy and traditional therapy are associated with better therapeutic results and less drug resistance [4]. Identifying small molecules that target oncogenes and/or key regulatory proteins in CRC is a promising strategy for developing novel cancer therapies.

c‐Myc is indeed a well‐known oncoprotein implicated in various types of cancer, including colorectal cancer (CRC). It plays a crucial role in regulating cell proliferation, apoptosis, metabolism, and differentiation. Dysregulation of c‐Myc expression, often through overexpression, can lead to uncontrolled cell growth and contribute to the development and progression of cancer. Specifically, in CRC, overexpression of c‐Myc is frequently observed. Studies have shown that approximately 70% of CRC tumors exhibit increased levels of c‐Myc expression compared with normal tissue. This overexpression can drive tumor growth and metastasis in CRC, making c‐Myc an important target for therapeutic intervention in this disease [5]. c‐Myc promotes cell cycle progression through its effects on several downstream regulators, including Cyclins, CDKs, and cell cycle inhibitors. The c‐Myc protein dimerizes with Myc‐associated factor X (MAX) via the C‐terminal bHLH‐LZ domain before interacting with E‐box (CACGTG) regions [6, 7]. c‐Myc levels are tightly controlled by posttranslational modifications, especially ubiquitination, which mediates its proteasomal degradation. Under normal conditions, the c‐Myc protein has a short half‐life of at most 20–30 min [8]. However, increased accumulation of the c‐Myc protein is found in cancers due to increased protein stability and impaired protein turnover [9, 10]. Phosphorylation at serine 62 (Ser62) and threonine 58 (Thr58) are the key regulatory events in c‐Myc stabilization and degradation. Phosphorylation at Ser62 promotes the activity of Myc dimers [11]. Moreover, it is also required for further phosphorylation of c‐Myc at Thr58, which is associated with its ubiquitin‐mediated proteasomal degradation [12, 13, 14]. Phosphorylation at Ser62 is indeed associated with target gene activation through increased dimerization with its partner MAX, and it also prolongs the c‐Myc half‐life [15]. Therefore, several effective molecular candidates have been developed that target mechanisms of c‐Myc destabilization and degradation [16, 17]. However, there are only a few drugs that directly target c‐Myc. Therefore, we focused on identifying a small molecule that could be a candidate c‐Myc inhibitor in order to provide a better therapeutic option for patients.

Benzoxazine dimer derivatives are small‐molecule chemical compounds that are synthesized via a two‐step method: the Mannich reaction and ring‐opening dimerization [18]. Some of these derivatives were previously reported to have anticancer effects, including apoptosis induction, autophagy‐mediated induction of apoptotic cell death, and metastasis inhibition in non‐small‐cell lung cancer [19, 20, 21]. EMD was reported to be a c‐Myc‐targeting compound that induced c‐Myc proteasomal degradation [19]. The structure of ECD is modified from that of EMD, with the N‐substituent a cyclohexyl group instead of a p‐methyl group [18]. Consequently, this research focused on the anticancer activities of the modified structural compound, ECD, by studies in *in vitro*, *in silico*, and *in vivo* models, and the ability of ECD to inhibit the c‐Myc oncoprotein in colorectal cancer was evaluated.

## 2 Matherials and methods

### 2.1 Materials

Roswell Park Memorial Institute (RPMI) 1640 medium, fetal bovine serum (FBS), phosphate‐buffered saline (PBS), 0.25% trypsin–EDTA, and dimethyl sulfoxide (DMSO) were obtained from PAN Biotech (Aidenbach, Germany). Penicillin–streptomycin was obtained from Gibco (Grand Island, NY, USA). Bovine serum albumin (BSA) and skim milk powder were obtained from Carl Roth (Karlsruhe, Germany). Hoechst 33342 was purchased from Sigma–Aldrich Co. (St. Louis, MO, USA). Primary antibodies against c‐Myc (#5605), PARP (#9532), Cyclin D1 (#2922), p21 (#2946), p‐Ser62‐c‐Myc (#13748), p‐Thr58‐c‐Myc (#46650), as well as a biotinylated protein ladder (#7727) and an anti‐biotin, HRP‐linked antibody (#7075), were acquired from Cell Signalling Technology (Danvers, MA, USA). The anti‐H2AX (#07–627) and anti‐γ‐H2AX (#05–636) primary antibodies were purchased from Merck Millipore (HES, Germany). The anti‐Cyclin B1 primary antibody (#sc‐752) was purchased from Santa Cruz Biotechnology, while the anti‐p‐Chk‐1 primary antibody (#ab59239) was obtained from Abcam. The anti‐H3K9me3 (#39765) primary antibody was obtained from Active Motif (Waterloo, Belgium), and the anti‐GAPDH (#MAB5476)‐HRP primary antibody was obtained from Abnova (Taipei, Taiwan). The goat anti‐rabbit IgG (#31460) and goat anti‐mouse IgG (#31430) secondary antibodies used for western blot analysis, the Alexa Fluor 488‐conjugated goat anti‐rabbit IgG (#A11034) secondary antibody used for immunocytochemical staining, and Alexa Fluor™ 555‐conjugated phalloidin were purchased from Thermo Fisher Scientific (Waltham, MA, USA). Primary antibodies for immunohistochemical analysis against p21 Waf1/Cip1 (#DCS60), γ‐H2AX (#JBW301), Pan‐CK (AE1/AE3), c‐Myc (#EP121), and Ki‐67 (#Mib1) were provided by Cell Signalling Technology (Danvers, MA, USA), Merck Millipore (Burlington, MA, USA), Zytomed (Berlin, Germany), Epitomics (CA, USA), and Dako (Nowy Sącz, Poland), respectively. All chemicals were of analytical grade and used as received. EMD and ECD were obtained from the Department of Materials Engineering, Faculty of Engineering, Kasetsart University, Thailand. All fertilized chicken eggs sourced from Lohmann (Ankum, Germany) are from the chicken strain “Lohmann Brown‐Classic.”

### 2.2 Syntheses of dihydro‐benzoxazine dimers

**Fig. 1:**
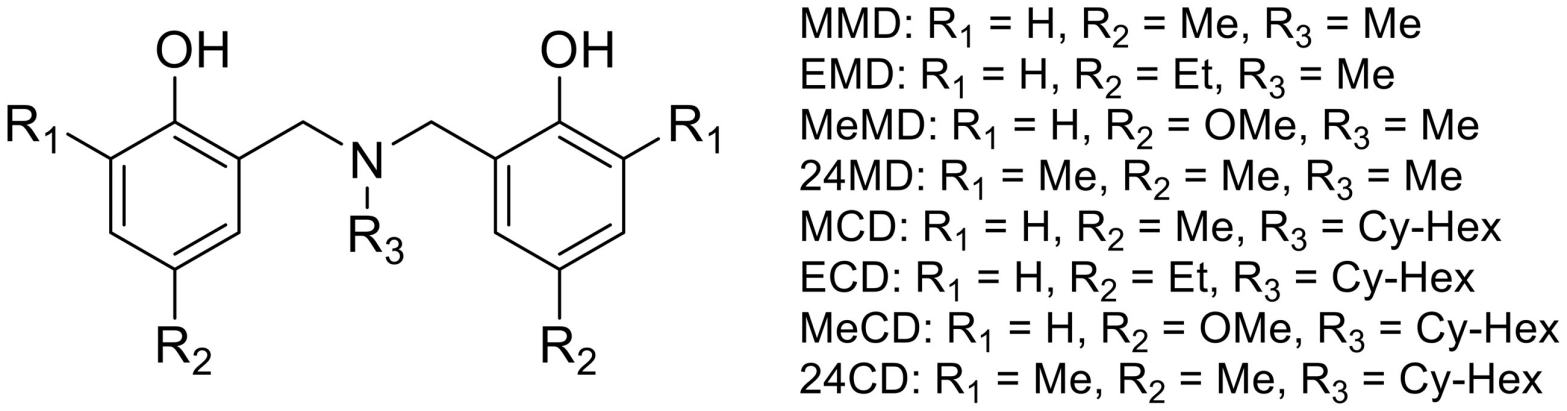
Synthesis of the benzoxazine dimers via the Mannich reaction and subsequent ring‐opening reaction. The synthesized compounds consist of 2, 2′‐((methylazanedyl) bis (methylene)) bis (4‐methylphenol); MMD, 2, 2′‐((methylazanedyl) bis (methylene)) bis (4‐ethylphenol); EMD, 2, 2′‐((methylazanedyl) bis (methylene)) bis (4‐methoxyphenol); MeMD, 6, 6′‐((methylazanedyl) bis (methylene)) bis (2, 4‐dimethylphenol); 24MD, 2, 2′‐((cyclohexylazanedyl) bis (methylene)) bis (4‐methylphenol); MCD, 2, 2′‐((cyclohexylazanedyl) bis (methylene)) bis (4‐ethylphenol); ECD, 2, 2′‐((cyclohexylazanedyl) bis (methylene)) bis (4‐methoxyphenol); MeCD and 6, 6′‐((cyclohexylazanedyl) bis (methylene)) bis (2, 4‐dimethylphenol); 24CD.

Dihydro‐benzoxazine dimers were synthesized through a two‐step process: (a) a Mannich reaction to prepare the benzoxazine monomers, and (b) a ring‐opening reaction involving the benzoxazine monomers and phenols. In the Mannich reaction, primary amines (150 mmol), paraformaldehyde (300 mmol), and para‐substituted (or ortho‐ and para‐substituted) phenols (150 mmol) were dissolved in 100 mL of dioxane and refluxed for 6 hours. Upon completion, dark‐yellow solutions were obtained. The solvent was then removed under reduced pressure using a rotary evaporator. To eliminate unreacted starting materials and impurities, the solutions were extracted with 3 N NaOH and deionized water, with dichloromethane added before liquid–liquid extraction. The extracted products were dried over anhydrous sodium sulfate, and the dichloromethane was evaporated. This resulted in viscous brown liquids containing the benzoxazine monomers. Equal molar amounts of the benzoxazine monomers and phenols were mixed without solvent, and the mixture was stirred at 60 °C for 24 h until it solidified. Diethyl ether was then added to remove impurities, and the dihydro‐benzoxazine dimers were isolated as white precipitates. The chemical structures of the synthesized dihydro‐benzoxazine dimers are shown in Fig. 1. The characterization data reported in the SI are in line with the literature [18, 22, 23, 24, 25, 26]. The ^1^H NMR and ^23^C NMR profiles of each synthetic compound were reported in Fig. S6.

### 2.3 Computational c‐Myc modeling and molecular docking

The X‐ray structure of the c‐Myc/MAX complex was retrieved from the RCSB Protein Data Bank (PDB ID: 1NKP) [27]. The 3D structures of ECD were constructed using GaussView 5.0 and then optimized by Gaussian09 (Gaussian, Inc.: Wallingford, CT, USA, 2009) using the B3LYP/6‐31G(d) basis set. The AutoDockFR software suite [28] was used to prepare the protein and ligand data in PDBQT file format before molecular docking was performed using AutoDock Vina 1.2 [29]. The centroid of the binding pocket at the c‐Myc/MAX interface [30] was designated as the site for molecular docking simulation. All grid dimensions were set to 25 Å for the c‐Myc/MAX complex. The grid center coordinates *x*, *y*, and *z* for the c‐Myc/MAX complex were established with the values 69.0, 74.7, and 34.1, respectively. The pose with the lowest AutoDock Vina score was selected as the representative conformation of ECD bound to the c‐Myc/MAX complex interface. The binding patterns of both compounds and their interactions were analyzed with 2D interaction diagrams using Discovery Studio Visualizer (BIOVIA, San Diego, CA, USA) and as 3D visualizations using the UCSF Chimera program [31].

### 2.4 Preparation of EMD and ECD

EMD and ECD were prepared as 40 mm master stock solutions by dissolving them in DMSO and then diluting them to 6‐, 10‐, 15‐, and 20‐mm stock solutions. Solutions of the desired treatment concentrations were freshly prepared by preparing 200‐fold dilutions of the stock solutions in complete medium before treatment. The final concentration of DMSO in all solutions used for treatment did not exceed 0.5% *v/v*.

### 2.5 Cell culture

The CRC cell lines HT29 (RRID: CVCL_0320), HCT116 (RRID: CVCL_0291), SW480 (RRID: CVCL_0546), and LoVo (RRID: CVCL_0399) were cultured in Roswell Park Memorial Institute (RPMI) 1640 medium supplemented with 10% fetal bovine serum (FBS) and 1% penicillin–streptomycin. The CRC cells were cultured in a 37 °C humidified incubator with 5% carbon dioxide. All the colorectal cell lines were purchased from ATCC. The cells were subcultured when they reached 75% confluence. All cell lines underwent genotyping through Multiplex Cell Authentication by Multiplexion (Heidelberg, Germany). Furthermore, all cell lines were routinely tested and confirmed to be free of mycoplasma contamination.

### 2.6 Western blot analysis

After treatment under the indicated conditions, HT29, HCT116, SW480, and LoVo cells were collected with a cell scraper and lysed with urea lysis buffer supplemented with a protease inhibitor cocktail and a phosphatase/protease inhibitor for 1 h on ice. The lysates were sonicated with 15 pulses per second at an amplitude of 30%. Then, they were centrifuged at 15,200 ***g*** for 10 min at 4 °C. The cell debris was discarded, and the supernatant was collected. The protein concentration was determined by using a DC™ Protein Assay Kit (Bio‐Rad, Munich, Germany). An equivalent amount of protein from each sample was diluted with lysis buffer and 6× loading dye before loading onto SDS/PAGE gels. The proteins were separated and transferred onto 0.2‐μm nitrocellulose membranes. The membranes containing the separated proteins were blocked with 5% nonfat milk in TBST (Tris‐buffered saline with 0.1% Tween containing 125 mm NaCl, 25 mm Tris–HCl (pH 7.5), and 0.1% Tween 20) for 1.5 h and incubated with a primary antibody overnight at 4 C with gentle agitation. Then, the membranes were incubated with an appropriate secondary antibody for 2 h at room temperature. Finally, the protein bands were detected using a chemiluminescent substrate and exposed to VWR® CHEMI only, Chemilumineszenzsystem (Avantor, PA, USA). Protein band densities were analyzed using the ImageJ software (version 1.52; National Institutes of Health, Bethesda, MD, USA).

### 2.7 Immunoprecipitation assay

HT29 and HCT116 cells were pretreated with 10 μm MG132 for 1 h and then treated with 75 μm EMD and ECD for 6 h. The treated cells were collected and lysed with urea buffer as described in the “western blot analysis” section. Magnetic beads provided in the Dynabeads Protein G Immunoprecipitation Kit (Thermo Fisher Scientific Inc., Waltham, MA) were washed with a washing buffer and incubated with a primary antibody (Ab) in binding buffer for 10 min with gentle agitation. The protein lysate was mixed with the bead–Ab complexes at 4 °C overnight. Then, the bead–Ab–antigen complexes were washed three times with 200 μL of washing buffer. The supernatant was removed, and an elution buffer was added to elute the Ab–antigen complexes from the beads. Western blot analysis was performed to evaluate protein ubiquitination.

### 2.8 Cell viability

For cytotoxicity assays, 1.0 × 10^4^ to 1.5 × 10^4^ cells per well were seeded in 96‐well plates for overnight incubation at 37 °C with 5% carbon dioxide. Then, the cells were treated with various concentrations of ECD (0, 30, 50, 75, and 100 μm). After 48 h, the cells were washed with 1× PBS and stained with crystal violet. The stain was solubilized with acetic acid for 15 min at room temperature with gentle agitation. The absorbance of crystal violet was measured at 595 nm with a VICTOR3 Multilabel Plate Reader (PerkinElmer, CT, USA). To quantify cell viability, the absorbance of the treated cells was divided by the absorbance of the untreated cells, and the data are reported as percentages of viable cells. The half‐maximal inhibitory concentrations (IC50) were calculated from the percentage of viable cells via the GraphPad Prism 9 program.

### 2.9 Chorioallantoic membrane (CAM) assay

All chick embryo chorioallantoic membrane (CAM) experiments were performed in compliance with the EU directive 2010/63 and were purchased from the certified breeding company Lohmann (Ankum, Germany). All breeding, housing, and handling of the parent flocks are conducted under certified conditions that meet EU animal welfare regulations. No animal license number is applicable, as the CAM assays were all terminated at EDD14 and are therefore not classified as animal experiments under EU Directive 2010/63.

The CAM xenograft assay was performed as previously described. [32, 33] Briefly, fertilized eggs were incubated in a shaking incubator for 7 days before dropping the CAM and opening a window on embryonic day (ED)8 in the eggshell. HT29 and HCT116 cells were seeded into 10‐cm dishes and expanded overnight. Then, the cells were treated with various concentrations of ECD (0, 50, and 75 μm) for 48 h. After that, the 1 × 10^6^ of surviving cells were collected and resuspended in 1:1 complete medium and Matrigel (Corning®). The Matrigel pellets were solidified in an incubator for at least 45 min but not more than 1 h. The pellets were loaded onto the CAM on Day 9, and incubation was continued for an additional 5 days in a standard incubator at 37 °C. After 5 days at ED14, the former tumors were harvested. A schematic illustration of the CAM assay workflow is shown in Fig. S2.

The percentage of tumors formed under each condition in the surviving eggs, and the tumor volume was calculated by the following equations:

$$\begin{array}{l} \text{Percentage of tumor forming}\\ \;=\frac{\text{number of tumor in each condition}}{\text{number of survived}\;\mathrm{egg}\;\text{in each condition}}\times 100 \end{array}$$

*V* = 0.52 × *W* × *H* × *L* when *W* is width, *H* is height and *L* is length in mm.

The tumors were prefixed with 4% paraformaldehyde overnight at room temperature before being fixed with 10% formalin, dehydrated, and embedded in paraffin to prepare tissue blocks. The blocks were sliced into sections before being subjected to further immunohistochemical staining. After deparaffinization, hematoxylin and eosin (H&E) and immunohistochemistry (IHC) for p21 (1 : 100), γ‐H2AX (1 : 2000), Pan‐CK (1 : 40), c‐Myc (1 : 100), and Ki‐67 (1 : 100) were performed.

### 2.10 Flow cytometry with PI

This flow cytometry staining assay was conducted to determine the cell cycle distribution after compound treatment. HT29, HCT116, SW480, and LoVo cells were seeded at a density of 5.5 × 10^5^ cells per dish in 6‐cm dishes. After expansion overnight at 37 °C with 5% carbon dioxide, the cells were treated with various concentrations of ECD (0, 50, 75, and 100 μm) for 48 h. Untreated control cells were collected at 24 and 48 h to normalize the cell cycle arrest rate due to the high confluence of the cells after 48 h. The cells were fixed with 70% ethanol on ice overnight. The supernatant was discarded, and the cells were extracted by incubation with extraction buffer at room temperature for 5 min. After that, the cells were stained with PI solution containing RNase in the dark at room temperature for 30 min. Excess dye was removed by washing with 1× PBS before analysis with a FACSCanto® II flow cytometer (BD Biosciences, CA, USA). The FlowJo™ software was used to quantify cell cycle distribution.

### 2.11 Immunocytochemical staining assay

HT29 and HCT116 cells were seeded on fibronectin‐coated glass coverslips in six‐well plates at a concentration of 0.2 × 10^6^ cells·mL^−1^ and incubated overnight at 37 °C with 5% carbon dioxide. The cells were treated with various concentrations of ECD (0, 50, and 75 μm) for 48 h. At the end of the incubation, the cells were fixed with 4% paraformaldehyde for 10 min at room temperature. Then, the cells were permeabilized with 0.1% Triton‐X for 7 min and blocked with 1% BSA for 30 min at 37 °C. Then, the cells were incubated with a primary antibody at a suitable concentration depending on the antibody for 1 h. After that, the cells were washed and incubated with secondary antibodies conjugated to a fluorophore at a 1 : 500 dilution in the dark for 1 h. Actin filaments and nuclei were stained with phalloidin‐555 and DAPI, respectively. The coverslips were stored at 4 °C in Fluoromount medium until imaging.

### 2.12 Sample preparation for shotgun proteomics

Cells were lysed in 0.5% SDS, vortexed, and centrifuged at 10 000 × ***g*** for 15 min. The supernatant was then subjected to acetone precipitation by adding two volumes of cold acetone and incubating overnight at −20 °C. The precipitated proteins were pelleted by centrifugation at 10 000 × ***g*** for 15 minutes, and the supernatant was discarded. The pellet was then dried and stored at −80 °C. *Prior* to analysis, the proteins were reduced with 10 mm dithiothreitol and alkylated with 30 mm iodoacetamide, both in 10 mm ammonium bicarbonate. Digestion was performed with sequencing‐grade porcine trypsin (1 : 20, w/w) for 16 h at 37 °C. The resulting peptides were concentrated by speed vacuum and reconstituted in 0.1% formic acid for LC–MS/MS analysis.

### 2.13 Liquid chromatography–tandem mass spectrometry (LC/MS–MS)

Samples were analyzed by nano‐liquid chromatography–tandem mass spectrometry (nanoLC–MS/MS) using an Ultimate3000 Nano/Capillary LC System coupled to an HCTUltra LC–MS system equipped with a nanoflow CaptiveSpray ion source. Five microliters of each tryptic peptide digest was enriched on a 300 μ‐Precolumn and then separated on a 75 μm I.D. × 15 cm Acclaim PepMap RSLC C18 column maintained at 60 °C. Peptide elution was achieved with a 30‐min gradient of 5–55% solvent B (0.1% formic acid in 80% acetonitrile) at a flow rate of 0.30 μL·min^−1^. Ionization was performed at 1.6 kV with nitrogen as the drying gas. Collision‐induced dissociation (CID) was carried out with nitrogen, and MS and MS/MS spectra were collected in positive ion mode at 2 Hz over an *m/z* range of 150–2200. The collision energy was set to 10 eV relative to the *m/z* value. All samples were analyzed in triplicate.

### 2.14 Bioinformatics and data analysis

LC–MS data were processed using DecyderMS [34, 35] with the *Homo sapiens* protein database sourced from UniProt. Searches allowed up to three missed cleavages, with carbamidomethylation of cysteine as a fixed modification and methionine oxidation as a variable modification. Protein abundances in each sample are reported as log2 values. Visualization and statistical analysis of LC–MS data—including partial least squares discriminant analysis (PLS‐DA) and differential abundance analysis (volcano plot, and heatmap)—were performed using the MetaboAnalyst software, applying a significance threshold of *P* < 0.05 [36]. Protein enrichment and functional analyses for differentially abundant proteins were executed with ShinyGO version 0.77 [37]. Proteins identified in both untreated control and ECD‐treated cells were compared using Venn diagrams (http://jvenn.toulouse.inra.fr/app/index.html). Differentially abundant proteins were categorized based on Gene Ontology (GO) terms associated with cellular processes such as “cell death” (GO:0008219) and “cell cycle” (GO:0007049), utilizing the Protein Analysis through Evolutionary Relationships (PANTHER) system. The STITCH database version 5 (http://stitch.embl.de/) was employed to investigate shared and predicted functional interaction networks between the identified proteins and small molecules [38].

### 2.15 Statistical analysis

The results are presented as the mean ± SEMs of at least three independent measurements. Multiple comparisons to identify statistically significant differences among multiple groups were performed with one‐way ANOVA, and individual comparisons were then performed with Scheffé's *post hoc* test, while two‐group comparisons were performed using an unpaired *t*‐test. All the statistical calculations were performed with the SPSS software version 28 (SPSS Inc., Chicago, IL, USA). A *P* value < 0.05 was considered to indicate statistical significance. Graphs were generated with the GraphPad Prism 9 program. The calculated *P* values cannot be interpreted as hypothesis tests but only as descriptive values.

## 3 Results

### 3.1 Prediction of the possible binding site for ECD‐related c‐Myc inhibition by computational modeling analysis

**Fig. 2:**
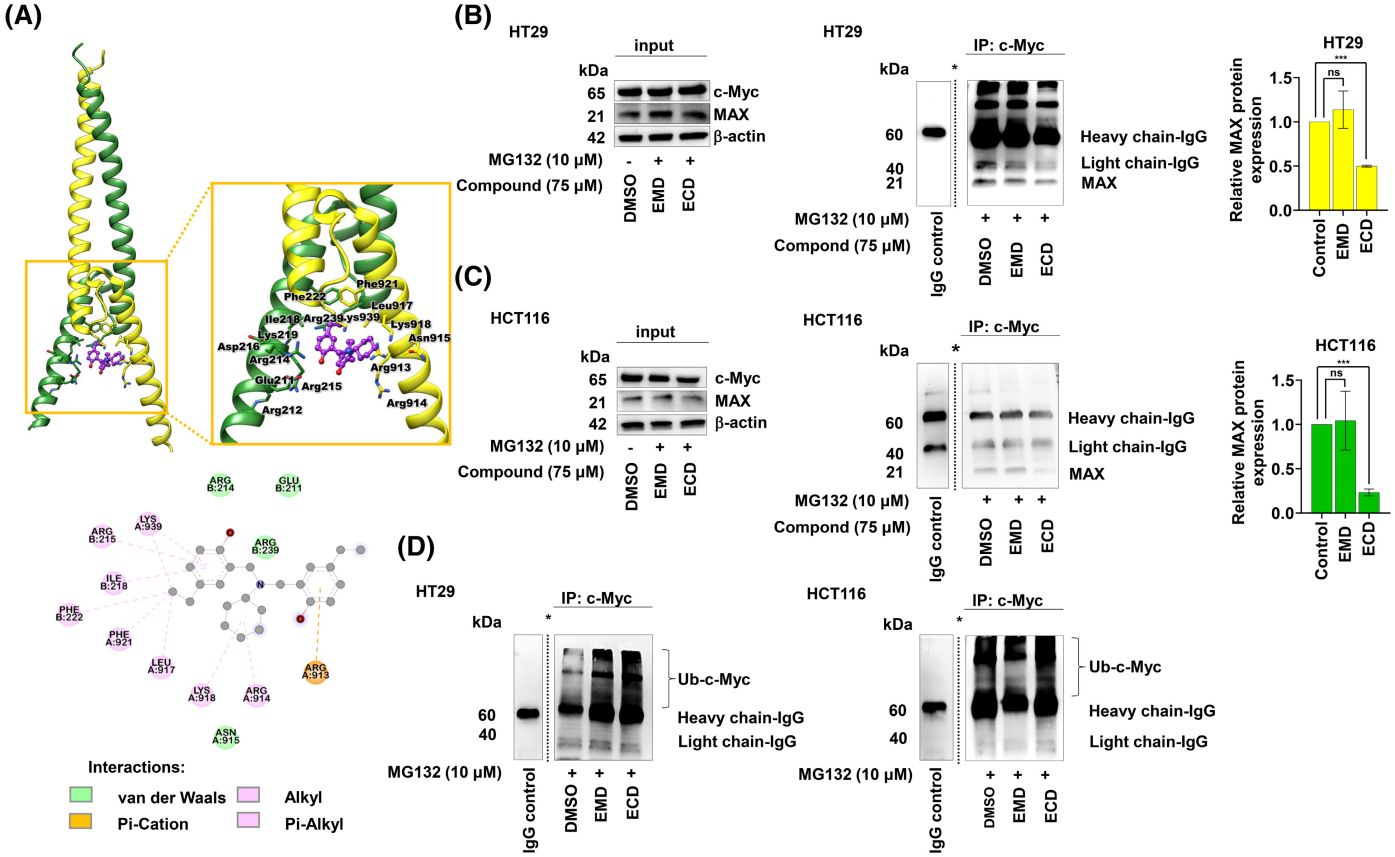
Indirect induction of c‐Myc degradation might be caused by disruption of the c‐Myc/MAX interaction. (A) Molecular docking analysis of ECD bound to the binding pocket formed by the interface between c‐Myc and Max. This molecular structure was shown in the graphical abstract. To reveal whether ECD disrupts the interaction between c‐Myc and MAX, protein lysates of HT29 cells were collected after pretreatment with MG132 (10 μm) followed by ECD treatment (75 μm). (B, C) Input protein lysates were measured to demonstrate the present of an interested protein target. GAPDH protein expression was evaluated to confirm the equal loading of each protein sample. The lysates were incubated with a mixture of beads and anti‐c‐Myc primary antibodies to pull down the c‐Myc/MAX protein complex. Then, the MAX protein level was measured by western blot analysis (*n* = 3). The densitometry of MAX protein level after c‐Myc pull‐down was demonstrated as a relative protein level. (D) HT29 and HCT116 cells were pretreated with MG132 for 1 h prior to treatment with 75 μm EMD or 75 μm ECD for 6 h. The protein lysates were collected and incubated with bead complexes conjugated to an anti‐c‐Myc antibody to pull down the protein of interest. Then, the ubiquitin protein level was evaluated by western blot analysis, and the data was presented in mean ± SEMs (*n* = 3). The statistical analysis of two‐group comparisons were performed using an unpaired *t*‐test (ns means not significant and *** *P* < 0.001 compared with the untreated controls). The * symbol represents the splicing out of additional bands.

We previously reported that EMD, a derivative of ECD, triggered c‐Myc protein degradation through ubiquitination [19]. Therefore, we hypothesized that, similar to EMD, ECD could also induce ubiquitin‐mediated proteasomal degradation of c‐Myc. Evading the activity of ligases, c‐Myc can avoid degradation and prolong its stability by interacting with the MAX protein, which is a highly stable protein [15]. Therefore, we investigated the mechanism by which ECD acts as a c‐Myc/MAX inhibitor. The interface between c‐Myc and MAX has been identified as a potential binding site for c‐Myc inhibitors [30, 39]. Consequently, ECD was docked into this well‐defined binding pocket, as depicted in Fig. 2A. The docking results indicated that ECD fit well into the binding interface of c‐Myc and MAX, forming hydrophobic interactions, including pi‐alkyl and alkyl interactions with several residues of c‐Myc (Arg914, Leu917, Lys918, Phe921, and Lys939) and MAX (Arg215, Ile218, and Phe222). Additionally, a pi‐cation interaction played a crucial role in binding the benzyl ring to Arg913 of c‐Myc. This finding supports the effect of ECD on blocking c‐Myc/MAX heterodimerization, leading to disruption of the stability of the complex and an increase in c‐Myc degradation. To confirm this prediction, coimmunoprecipitation (co‐IP) was performed to evaluate the abundance of the c‐Myc/MAX complex. Fig. 2B,C shows that the abundance of the c‐Myc/MAX complex was notably slightly decreased in HT29 and notably reduced in HCT116 cells, confirming that ECD can disrupt the c‐Myc/MAX complex or inhibit its formation.

In addition, disruption of the interaction between the c‐Myc and MAX proteins could increase c‐Myc proteasomal degradation. To confirm that ECD leads to c‐Myc destabilization, in turn leading to its ubiquitin‐mediated proteasomal degradation, the total c‐Myc, p‐c‐Myc^Ser62^, and p‐c‐Myc^Thr58^ levels were evaluated in CRC cells after ECD treatment, and EMD treatment was used as a comparator. Western blot analysis revealed that the levels of the protein markers in HT29 and HCT116 cells were greatly decreased. Remarkably, MG132, a proteasome inhibitor, was applied to maintain the level of the c‐Myc protein by protecting it from proteasomal degradation. The addition of MG132 clearly influenced the proportion of the phosphorylated form of c‐Myc. In HT29 cells, the p‐c‐Myc^Thr58^ level was notably increased, while the p‐c‐Myc^Ser62^ level was reduced. In contrast, in HCT116 cells, the p‐c‐Myc^Thr58^ level was not noticeably altered, while the p‐c‐Myc^Ser62^ level was only slightly changed (Fig. S1). We also tested our hypothesis regarding ubiquitin‐mediated c‐Myc degradation via co‐IP and evaluated the level of the c‐Myc–ubiquitin complex (polyubiquitin–c‐Myc) in HT29 and HCT116 cells after treatment with DMSO (vehicle) or 75 μm ECD or EMD for 3 h. Fig. 1D shows that the polyubiquitination of c‐Myc was conspicuously elevated after EMD or ECD treatment compared with that in the DMSO vehicle control group. Thus, ECD also mediates c‐Myc stability through inhibition of its ubiquitin‐mediated proteasomal degradation, similar to EMD.

**Fig. 3:**
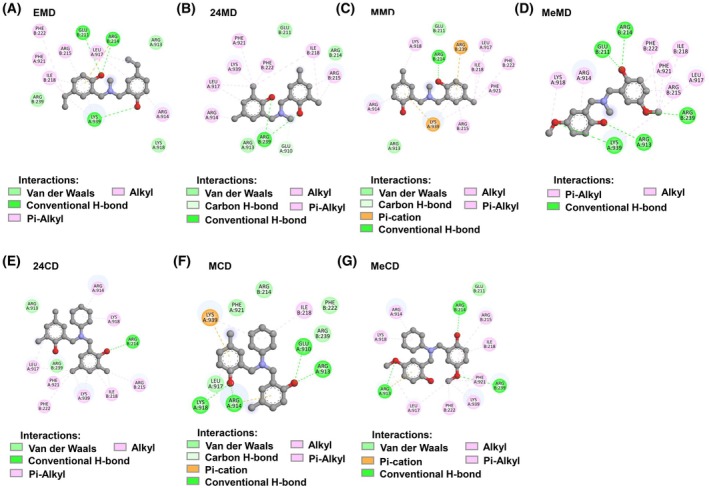
The binding interaction of benzoxazine dimer derivatives to the c‐Myc/MAX interacting complex (PDB ID: 1NKP). (A) The interaction between EMD and the c‐Myc/MAX complex, (B) the interaction between 24MD against c‐Myc/MAX complex, (C) the interaction between MMD against c‐Myc/MAX complex, (D) the interaction between MeMD against c‐Myc/MAX complex, (E) the interaction between 24CD against c‐Myc/MAX complex, (F) the interaction between MCD against c‐Myc/MAX complex, and (G) the interaction between MeCD against c‐Myc/MAX complex.

**Fig. 4:**
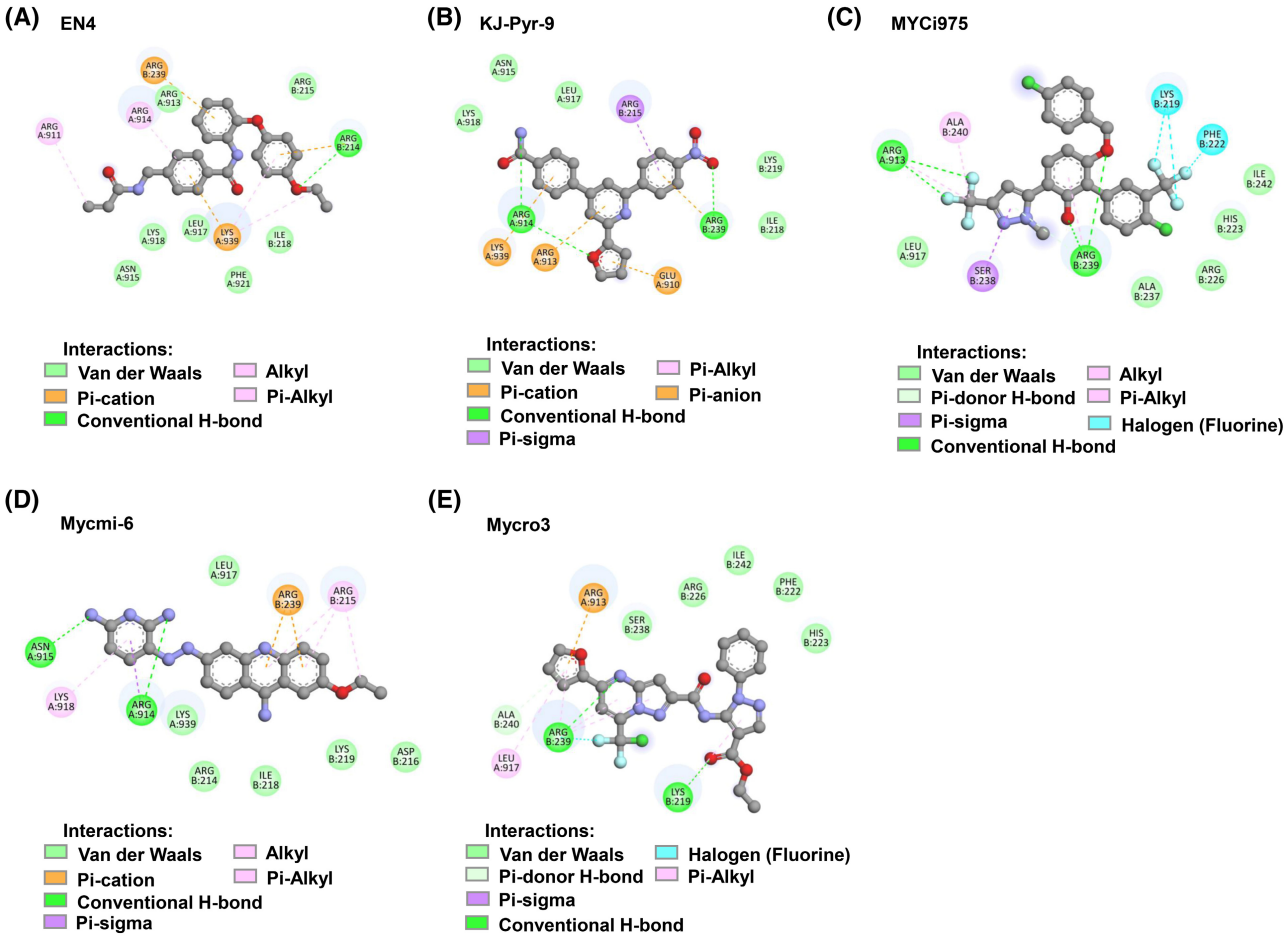
The binding interaction of Myc inhibitors to the c‐Myc/MAX interacting complex (PDB ID: 1NKP). (A) The interaction between EN4 and the c‐Myc/MAX complex, (B) the interaction between KJ‐Pyr‐9 and the c‐Myc/MAX complex, (C) the interaction between MYCi‐978 and the c‐Myc/MAX complex, (D) the interaction between Mycmi‐6 and the c‐Myc/MAX complex, and (E) the interaction between Mycro3 and the c‐Myc/MAX complex.

Moreover, we compared the binding energy of ECD with other known c‐Myc/MAX inhibitors that have been reported to inhibit tumor formation *in vivo*. The interacting bonds between each benzoxazine derivative and other myc inhibitors against the c‐Myc/MAX binding site were demonstrated in Figs 3, 4 and, respectively. As shown in Table 1, the ranges of the binding energies of ECD and the other reported inhibitors at the same interaction site did not differ significantly. Since the primary aim of this study was to improve benzoxazine dimer derivative for more effective targeting of the c‐Myc protein, the structural modification led to ECD demonstrating increased binding energy for a specific region of c‐Myc compared to EMD (Table 1). This finding represents a step toward identifying the key structural components necessary for activity optimization.

**Table 1:** Docking scores of benzoxazine derivatives and reported inhibitors toward the c‐Myc/Max complex obtained via AutoDock Vina scoring function.

Compound	Binding energy (kcal·mol^−1^)
ECD	−6.002
EMD	−5.387
24MD	−5.319
MMD	−5.623
MeMD	−5.328
24CD	−6.141
MCD	−6.314
MeCD	−5.860
EN4	−6.138
KJ‐Pyr‐9	−6.753
MYCi975	−6.609
Mycmi‐6	−6.261
Mycro3	−6.320

### 3.2 Proteomic analysis links ECD action with DNA damage

**Fig. 5:**
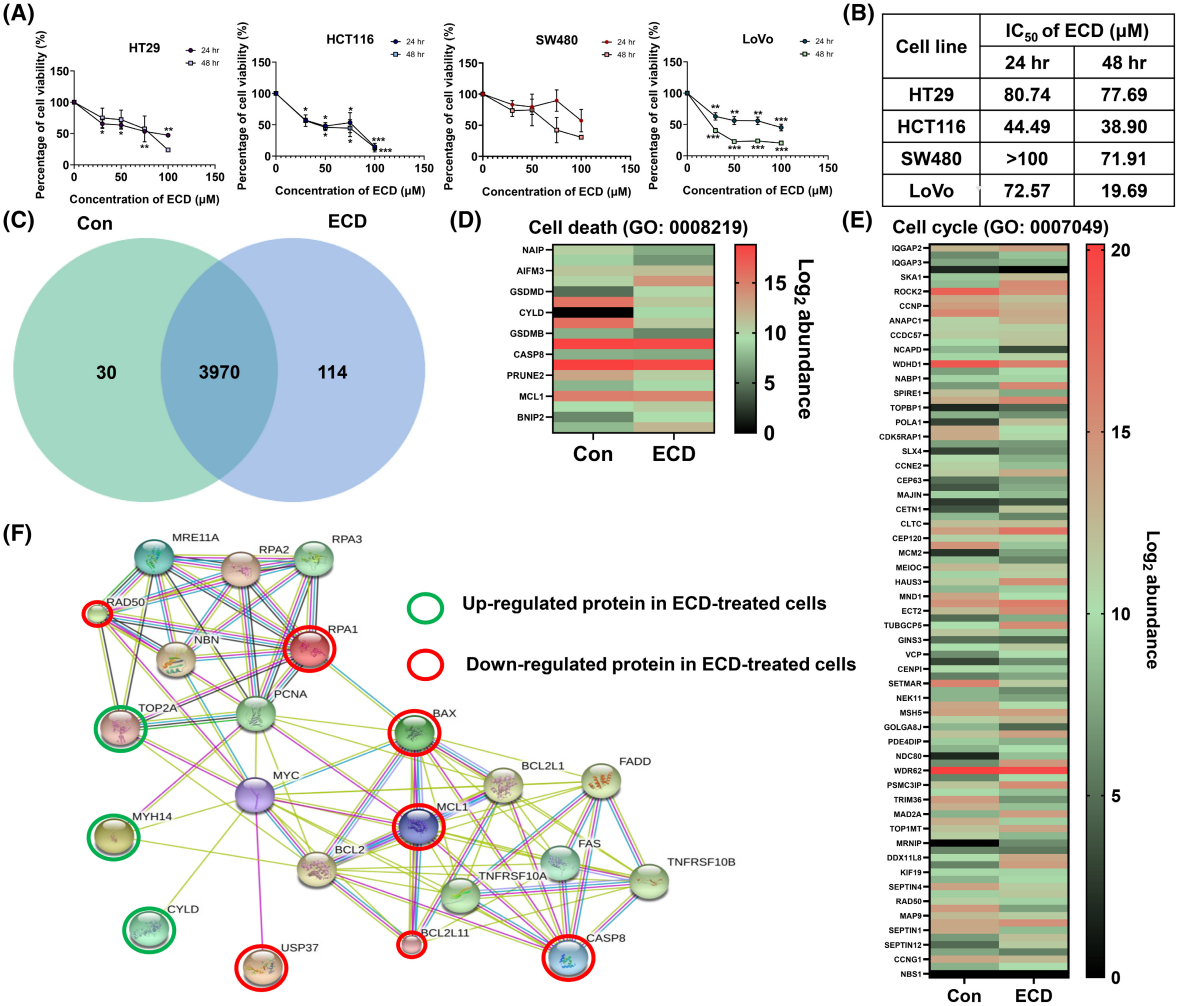
Alterations in protein expression after ECD treatment in HT29 cells. (A) Cell viability was evaluated by using crystal violet staining and measuring the absorbance at 570 nm after treatment with various concentrations of ECD (0–100 μm) for 24 and 48 h. (B) The IC_50_ of ECD in CRC cells was calculated via a crystal violet assay. The data are presented as the means ± SEMs (*n* = 3). The statistical analysis of multiple groups were performed with one‐way ANOVA, and individual comparisons were then performed with the Scheffe's *post hoc* test (* 0.01 ≤ *P* < 0.05, ** 0.001 ≤ *P* < 0.01 and ***P < 0.001 compared with the untreated controls). (C) Venn diagram showing the proteins expressed in control and ECD‐treated cells. (D, E) Heatmap showing the expression levels of proteins annotated to the terms “cell death” (GO:0008219) and “cell cycle” (GO:0007049). Expression from low to high is indicated by the color scale from green to red. Please see Tables S1, S2 for details of the proteins. (F) Protein–protein interaction network of the proteins expressed in untreated control cells and ECD‐treated cells. The red circles represent downregulated proteins, and the green circles represent upregulated proteins.

We evaluated the properties of ECD after an extended treatment time. First, the appropriate conditions for ECD treatment were examined in the following CRC cell lines: HT29 and SW480 cells, expressing mutant p53; and HCT116 and LoVo cells, expressing wild‐type p53. The cells were treated with various concentrations of ECD (0–100 μm) for 24 or 48 h. Cell viability was analyzed by a crystal violet assay. ECD significantly decreased cell viability in a concentration‐dependent manner in three of the cell lines—except for SW480—relative to the untreated control groups. These results demonstrated that more HCT116 and LoVo cells than HT29 and SW480 cells were killed by ECD (Fig. 5A). The half‐maximal inhibitory concentration (IC_50_) values are shown in Fig. 5B. The values were calculated from single values based on equations fitted to the pooled data.

The induction of DNA damage was revealed via proteomic analysis. Proteomics is the current ubiquitous approach for the identification of biomarkers with changes in expression in cells after exposure to cytotoxic compounds. This approach provides a clear representative picture of the biological processes occurring in the cells. To understand the mechanisms active within the cells after ECD treatment, we performed proteomic analysis of HT29 cells after treatment with or without 75 μm ECD for 24 h. LC–MS/MS‐based proteomic analysis identified 4000 and 4084 expressed proteins in control and ECD‐treated cells, respectively. The Venn diagram demonstrated that 3970 proteins overlapped between the groups, whereas 30 proteins were expressed specifically in the control group, and 114 proteins were expressed only in the ECD‐treated group (Fig. 5C–E).

The 4114 proteins identified in the whole‐proteome data were classified according to the GO term “cellular process” (GO:0009987) and then categorized according to the GO terms “cell death” (GO:0008219) and “cell cycle” (GO:0007049) via the PANTHER system. Among these proteins, 100 proteins were annotated to “cell cycle,” and 18 proteins were annotated to “cell death,” as shown in Fig. 5C–E and Tables S1, S2. Notably, MRNIP/MRE11A and CYLD were expressed in the ECD‐treated group but not in the control group, while RPA1 was expressed only in the control group.

The top 20 most upregulated and downregulated proteins annotated to GO:0008219 and GO:0007049 were selected for validation of their potential association with the c‐Myc protein using Search Tool for Interacting Chemicals 5.0 (http://stitch.embl.de/). Only proteins directly related to the c‐Myc protein are shown in Fig. 5F. The results showed that c‐Myc was directly related to TOP2A, MYH14, cylindromatosis (CYLD), and USP37. Moreover, it was also connected to MRNIP/MRE11A (MRE11‐RAD50‐NBS1) and RPA1. Interestingly, MRNIP was strongly upregulated when compared with control, whereas RAD50 was slightly downregulated, and NBS1 was not even detectable. TOP2A was enriched in ECD‐treated cells. Finally, the results of this *in silico* proteomic analysis were related to the ATM‐ or ATR‐mediated DNA damage response and the removal of toxic DNA adducts [40]. In this regard, it has been described that an attenuated TOP2 function let cytotoxic TOP2 protein–DNA covalent bound complexes persist [41]. These reports are well‐correlated with the observed increase in γ‐H2AX foci *in vitro* and *in vivo* upon ECD exposure (Fig.S3).

DNA damage induction is a fundamental mechanism that induces apoptotic cell death in response to numerous cancer treatments. Hence, we studied whether ECD causes DNA damage and triggers the DNA damage response as we interpreted the mode of mechanism from proteomic results. HT29 and HCT116 CRC cells were treated with various concentrations of ECD (0–100 μm) for 48 h. A concentration‐dependent increase in DNA damage was observed by immunocytochemical analysis of γ‐H2AX, a well‐known marker of DNA damage (Fig. S3A). These findings were confirmed by western blot analysis, where the γ‐H2AX levels were remarkably increased in HT29 cells and moderately changed in HCT116 cells, while the total H2AX levels were not altered in either CRC cell line (Fig. S3B,C). Next, immunohistochemical staining for γ‐H2AX in formalin‐fixed, paraffin‐embedded (FFPE) tumors harvested from the CAM model was performed. In contrast to the western bloting where adherent alive and swimming dead cells were analyzed at the same time, the CAM model approach illuminates only the surviving adherent cells. HT29 and HCT116 CRC cells were treated with various concentrations of ECD (0–75 μm) for 48 h before adherent cells were transplanted onto the CAM. The immunohistochemical score was calculated based on the percentage of stained cells per unit area. The histochemical score was calculated, and the samples were classified into 4 groups, as shown in Fig. S3E,F. We suggest that the lower repair capacity of ECD‐treated cells might diminish their tumor‐forming capability, which we will demonstrate in further results.

### 3.3 ECD causes apoptotic cell death in CRC cells and significantly decreases the G1 subpopulation

**Fig. 6:**
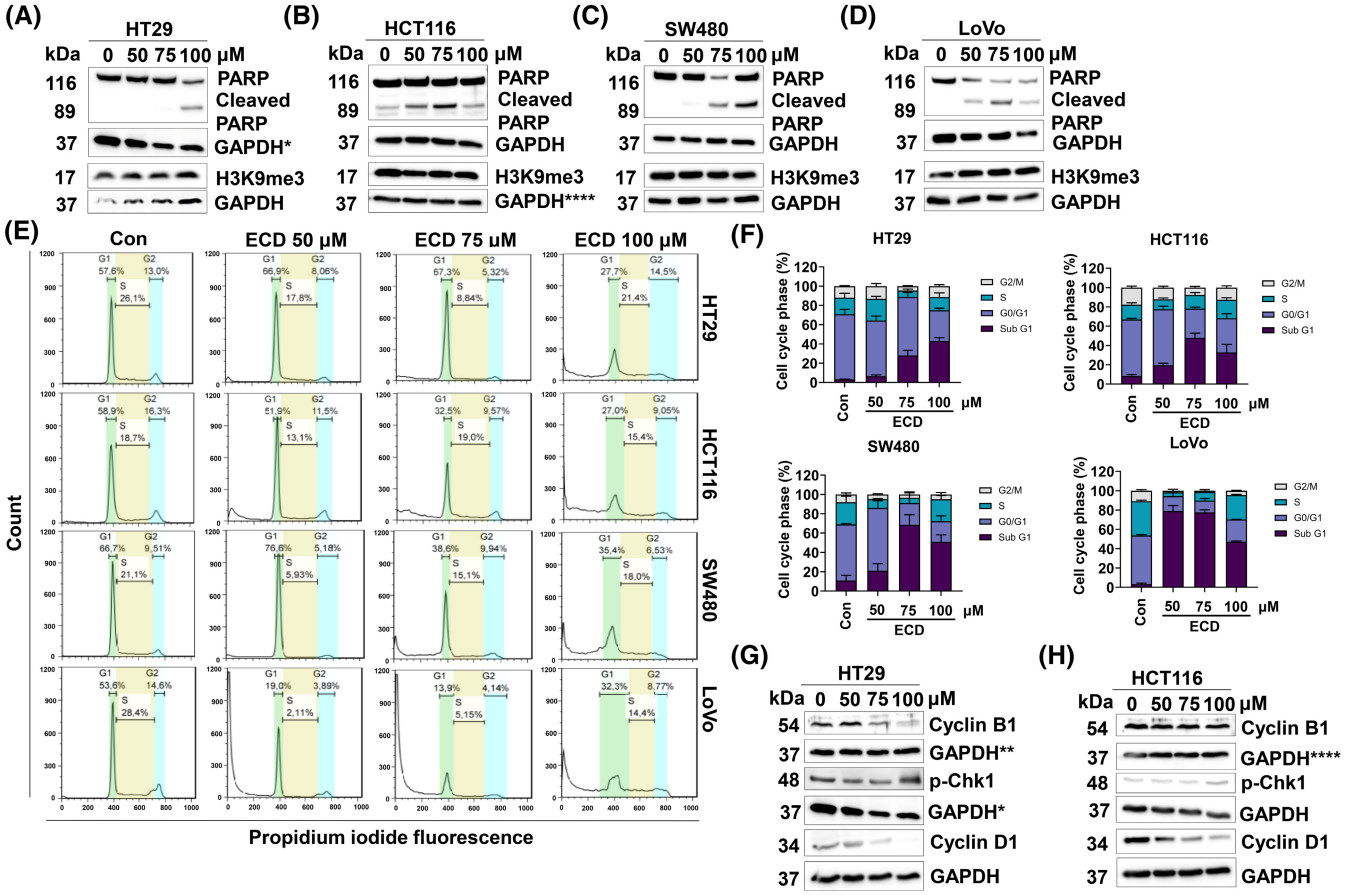
ECD induces apoptotic cell death in HT29, SW480, HCT116, and LoVo CRC cells. (A–D) Western blot analysis was performed to measure the levels of related apoptosis and cellular senescence markers. GAPDH protein expression was evaluated to confirm the equal loading of each protein sample. (*n* = 3). (E, F) CRC cells were treated with various concentrations of ECD (0–100 μm) for 48 h and then stained with PI. The cell cycle distribution was evaluated by flow cytometry, and the percentages of cells in each phase of the cell cycle are presented. The data are presented as the means ± SEMs (*n* = 3). (G, H) The cell cycle checkpoint markers were evaluated by western blot analysis. CRC cells were treated with various concentrations of ECD (0–100 μm) for 48 h before being used for western blot analysis. GAPDH protein expression was evaluated to confirm the equal loading of each protein sample (*n* = 3). The GAPDH bands with the *, ** and **** symbols indicate the bands used for normalization of other protein markers in further figures. The GAPDH with * and **** symbol in (A) and (B) were repeatedly used in (G) and (H), respectively. GAPDH**** was also applied again in Fig. S3C.

PARP is a key factor for the repair of damaged DNA. It is cleaved by an apoptotic enzyme called caspase. Therefore, the cleavage of PARP is considered a hallmark of apoptosis [42]. The large increase in the level of cleaved PARP in ECD‐treated cells indicated that treatment with ECD at a toxic concentration strongly induced apoptotic cell death in all CRC cell lines (Fig. 6A–D). Since the level of H3K9me3 did not change in any of the cell lines after ECD treatment for 48 h, we suggested that ECD treatment did not induce senescence in CRC cells. These results were confirmed by flow cytometry with PI staining. After treatment with various concentrations of ECD (0–100 μm) for 48 h, the percentage of sub‐G1 phase cells, representing dead cells with DNA fragmentation, was increased (Fig. 6E,F), confirming that cell death was induced by ECD in CRC cells relative to the DMSO vehicle control‐treated cells.

Interestingly, there was no induction of a cell cycle arrest in HT29 and HCT116 CRC cells. Whereas the G2/M and the S‐phase cell subpopulations did not change, the percentage of cells in G1 significantly decreased in all four CRC cell lines (Fig. 6E,F). Obviously damaged cells exit the cell cycle in M‐phase. Indeed, a faulty G2 control point was evident in western blotting for HT29 and HCT116 cells treated with various concentrations of ECD (0–100 μm) for 48 h (Fig. 6G,H). The levels of p‐Chk1 and Cyclin B1 proteins, which are involved in cell cycle delays to allow DNA repair, did not change significantly at 50 μm and 75 μm ECD, suggesting a missing G2/M checkpoint. Only at 100 μm ECD did this checkpoint appear to be activated slightly (Fig. 6G,H). Cyclin D1 levels, that are crucial for the G1/S transition, were strongly decreased in both cell lines, corroborating the cell cycle PI analysis (Fig. 6G,H).

### 3.4 ECD suppresses c‐Myc expression and inhibits tumorigenesis capability *in vivo* via CAM assay

**Fig. 7:**
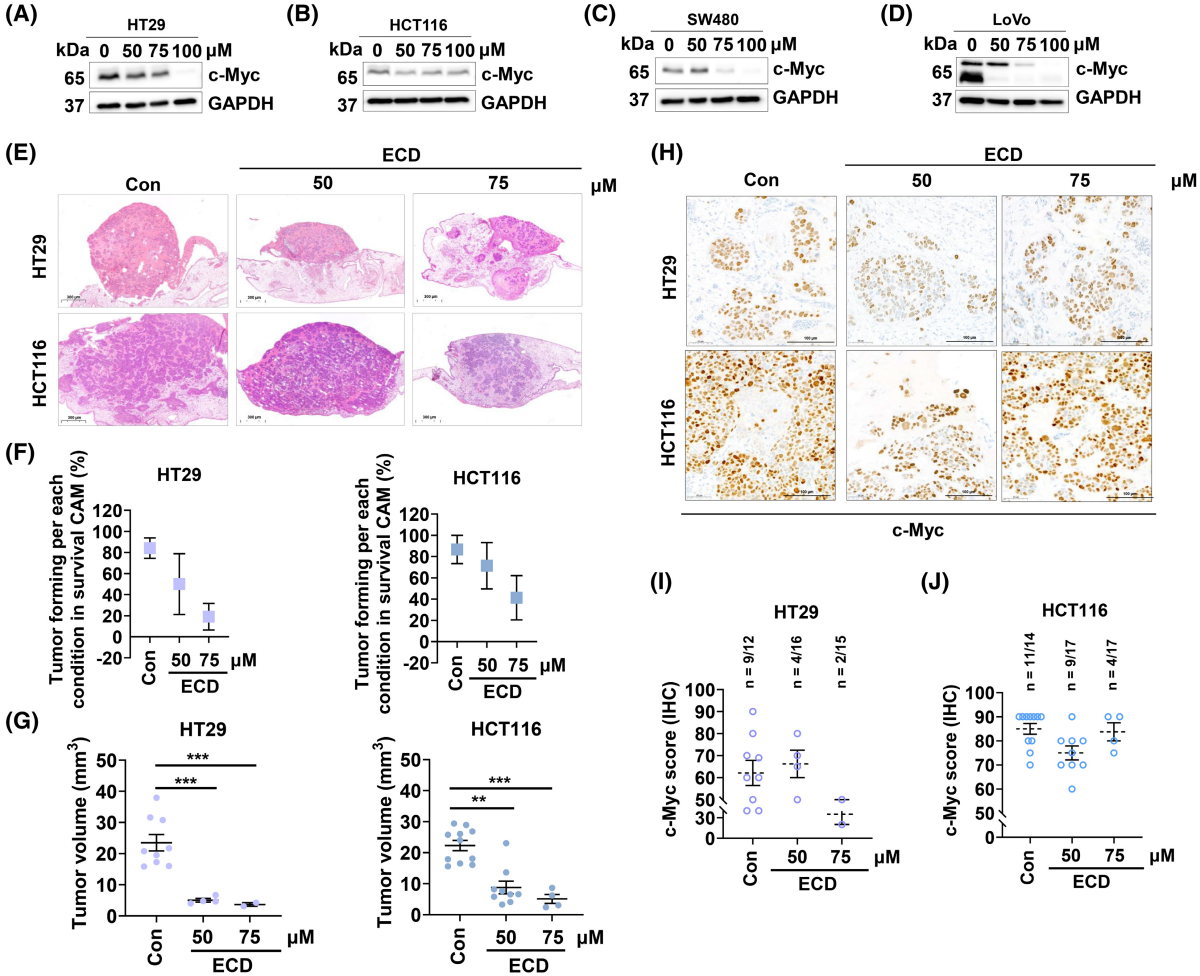
ECD inhibits proliferation and leads to apoptosis of CRC cells by reducing the level of nuclear c‐Myc. (A‐D) HT29, HCT116, SW480 and LoVo CRC cells were treated with various concentrations of ECD (0–100 μm) for 48 h before being used for western blot analysis. GAPDH protein expression was evaluated to confirm the equal loading of each protein sample (*n* = 3). (E) H&E‐stained sections of CAM microtumors. For this assay, HT29 and HCT116 CRC cells were treated with various concentrations of ECD (0–75 μm) for 48 h. Then, the adherent cells were transplanted onto the CAM. The tumors were collected after 5 days of incubation, and the xenografts were subjected to standard histological and immunohistochemical analyses. The scanned images of CAM sections were acquired at 4\u00D7 magnification (overview sections, scale bar: 200 μm) (*n* = 7). (F) The tumorigenic capability was quantified, and the results are presented as the percentage of tumors formed under each condition in the surviving eggs in the CAM assay. (G) The volume of each tumor is shown (*n* = 7). (H) The protein expression of c‐Myc was revealed by immunohistochemical staining of FFPE tumor tissues harvested from the CAM. The scanned images of CAM sections were acquired at 30× magnification (overview sections, scale bar: 50 μm) (*n* = 7). (I, J) The c‐Myc scores are determined by IHC staining. Con and ECD‐treated tumor sections determined by IHC staining were compared. The data are presented as the means ± SEMs (*n* =7). The statistical analysis of two‐group comparisons was performed using an unpaired *t*‐test (** 0.001 ≤ *P* < 0.01 and ***P < 0.001 compared with the DMSO vehicle control group).

To explore the mode of action of ECD in the suppression of c‐Myc‐related downstream processes, c‐Myc and its downstream targets were also evaluated after treatment for 48 h. As expected, the c‐Myc protein level was dramatically decreased in all cell lines in western blot analysis (Fig. 7A–D). To verify the antitumor effect of ECD *in vivo*, xenografts formed from HT29 and HCT116 cells were analyzed using a CAM model. The cells were treated with various concentrations of ECD (0–75 μm) for 48 h, and only adherent cells were collected and transplanted into 9‐day‐old chicken embryos. A diagram illustrating the experimental process for the CAM model is given in Fig. S2. The results showed that compared with that of the DMSO vehicle control‐treated cells, the tumor‐forming capability of the ECD‐treated HT29 and HCT116 cells was dramatically diminished, as shown by the percentage of tumors under each condition in the surviving embryos (Fig. 7E,F). Correspondingly, compared with that in the DMSO vehicle control groups, the volume of both the HT29 and HCT116 tumors that formed in the CAM was significantly decreased in the ECD‐treated groups (Fig. 7G).

In addition, our approach allowed for a further in‐depth evaluation of the long‐term effect on c‐Myc after stopping ECD exposure. For this, we analyzed CAM xenografts from HT29 and HCT116 cell lines by immunohistochemistry using a c‐Myc antibody. Although there was a tendency of decrease, the H‐scores of the c‐Myc marker were not significantly altered compared with those in the vehicle control groups (Fig. 7H–J). According to the number of tumors formed, it was found that even though the c‐Myc was still discovered in the treatment group, most of the cancer cells lost their tumorigenic capability. The corresponding Ki67 and Pan‐CK staining were provided in Fig. S4. Moreover, we have reported recently that the upregulation of cytoplasmic p21 might be the compensating mechanism that led the colorectal cancer cells to become resistant to 5FU treatment [43, 44]. After ECD treatment (0–100 μm) for 48 h, it was found that p21 was upregulated in HT29 but not in HCT116 (Fig. S5A,B). So, we also confirmed that the upregulation of p21 was not cytoplasmic p21. Notably, the histochemical staining of p21 revealed that p21 was mostly located in the cytoplasm (black arrow) rather than the nucleus (red arrow) in ECD‐treated HT29 tumors compared with the DMSO vehicle control‐treated tumors, but this localization pattern was not seen in HCT116 tumors (Fig. S5C). Even the H‐scores of cytoplasmic and nuclear p21 are presented in Fig. S5D,E. The results demonstrated that there were no significant differences in the levels of cytoplasmic and nuclear p21 between DMSO vehicle control‐treated cells and ECD‐treated cells. Even though p21 is a cell cycle inhibitor and tumor suppressor, it can act as an oncogene. Its function depends on the localization of the protein. p21 strongly disrupts cell cycle progression when it is in the nucleus. In contrast, it plays a role in cellular resistance when it is located in the cytoplasm. Because HT29 cells have been reported to be more resistant, the higher level of cytoplasmic p21 was not surprising. Obviously, ECD does not trigger this mode of cell escape.

## 4 Discussion

Benzoxazine dimer derivatives are compounds composed of a benzene ring connected to a dihydro‐oxazine heterocyclic ring. A series of reports have shown the anticancer activity of benzoxazine dimer derivatives via different mechanisms [19, 20, 21, 45]. ECD is a benzoxazine dimer derivative synthesized from 4‐ethylphenol, methylamine, and formaldehyde through the Mannich reaction followed by ring‐opening dimerization [46, 47]. ECD has a cyclohexyl group on the tertiary amine nitrogen instead of a p‐methoxyl group like the previously reported anticancer compound EMD (Fig. 1).

c‐Myc serves as a master regulator of proliferation since it is a multifunctional transcription factor that is involved in a large number of proliferative pathways [48]. It participates in promoting the expression of cell cycle markers, such as Cyclin D family members, while inhibiting the expression of antiproliferation markers, such as p21 [49]. Therefore, c‐Myc expression is tightly controlled in normal cells. Unfortunately, the control of c‐Myc expression is lost in cancer cells [5]. The uncontrolled overexpression of c‐Myc leads to tumorigenesis. Moreover, c‐Myc can interact with low‐affinity binding promoters, resulting in overactivation of the targeted gene [50]. Therefore, many studies have aimed to identify potential c‐Myc inhibitors [51]. Emerging evidence has demonstrated the application of c‐Myc‐targeted therapies for cancer, some of which have already gone through the clinical phase [52, 53]. c‐Myc is considered an undruggable protein due to its structural disorder; however, it attains stability upon binding with cofactors such as MAX. Consequently, the design of small molecules to indirectly inhibit c‐Myc presents a promising and more suitable approach [17]. Several therapeutic strategies for targeting c‐Myc, including transcriptional and translational inhibition, protein destabilization, and prevention and disruption of c‐Myc/MAX dimerization, have been investigated [54]. Inhibition of the c‐Myc/MAX complex is a promising therapeutic approach for CRC. This complex plays a crucial role in activating gene expression through binding to E‐box regions in promoters and thus controlling various cellular processes involved in cancer development and progression [55]. MAX can also form a homodimer, but it lacks a transcription activation domain even if it can interact with E‐box sequences [56]. Therefore, the stabilization of the MAX homodimer also antagonizes c‐Myc‐driven transcription [57]. Mad is the other MAX dimerization partner that antagonizes the function of the c‐Myc/MAX complex by active repression of transcription [58]. Even so, there are several c‐Myc/MAX inhibitors, but none of them have moved forward into clinical trials. These small molecules face several problems such as side effects, stability, and potency [55]. In this study, we investigated the potential of a benzoxazine dimer derivative as a c‐Myc‐targeted molecule.

Disruption of the interaction between c‐Myc and its binding partner MAX can indeed lead to the degradation of c‐Myc. This degradation can occur through several pathways, including ubiquitin–proteasome‐mediated degradation, where c‐Myc is tagged with ubiquitin molecules and targeted for degradation by the proteasome machinery [15, 55, 59]. In this study, we predicted the site of interaction between ECD and the c‐Myc/MAX complex (Fig. 2A). The co‐IP results confirmed the mode of disruption of the c‐Myc/MAX complex (Fig. 2B,C). ECD functioned as a potential inhibitor of the c‐Myc/MAX complex, and its binding energy score was within the same range as that of the previously reported inhibitors (Table 1). Moreover, ECD promoted an increase in c‐Myc^Thr58^ phosphorylation (Fig. S1) and the accumulation of ubiquitin‐conjugated Myc in ECD‐treated cells when MG132 was used as a proteasome inhibitor (Fig. 2D).

Comparing binding energy between benzoxazine dimer derivatives (Table 1), it was found that the compounds which contain *N*‐cyclohexyl moiety, ECD, 24CD, MCD, and MeCD, demonstrate a higher binding energy score than the compound which contains an *N*‐methyl group, EMD, 24MD, MMD, and MeMD. In addition, adding a methyl group at position 4 on the benzene ring might slightly increase affinity when compared to ethyl or methoxy moieties. The presence of a methyl group at position 2 on the benzene ring could give worse binding energy when compared to the absence of this group.

One challenge in developing small molecules is the off‐target effect, which may reduce efficacy and increase toxicity and side effects [60]. The benzoxazine dimer derivatives examined in this study could provide a foundation for developing compounds with enhanced specificity as c‐Myc inhibitors. ECD is a small molecule that can be synthesized and modified with relative ease. Additionally, the chemical reaction used to synthesize the benzoxazine dimer is straightforward, which allows for manageable control over the product. Future research may incorporate Fragment‐Based Drug Discovery (FBDD) to identify substituents with increased specificity for the c‐Myc b‐HLH‐LZ region. FBDD may be used to screen potential substituents, after which ECD could be modified or appended with groups that enhance binding to the target [61, 62, 63]. Figs 3, 4 and Table 1 indicate that molecules exhibiting higher binding energy to c‐Myc typically possess conventional hydrogen bonds and polar covalent bonds, particularly from hydroxyl and halogen groups. These interactions contribute to a stronger association between the molecules and the target binding site. Consequently, these findings may inform compound structure modification strategies to optimize fit at the binding site.

Beyond the specificity of configured interactions between compounds and their targets, appropriate dosing regimens and treatment intervals are also crucial factors. Currently, computer‐based modeling can be employed to predict the therapeutic window of emerging compounds. Administering compounds within the calculated therapeutic window may reduce off‐target effects by minimizing excess compound interactions with nontarget proteins. Additionally, scaffold‐hopping techniques have been implemented to enhance both the stability and selectivity of small molecules, further mitigating off‐target activity [64]. This technique may enable researchers to modify specific components of bioactive compound structures to enhance potency while preserving biological activity [65].

This development approach is consistent with the development pathways of several known protein inhibitors. For example, MYCi975 was developed from MYCi361 [66], while 10074‐G5 was further optimized into 3jc48–3 and JY‐3‐094 [67]. Similarly, Mycro3 evolved from Mycro1 and Mycro2 [68]. Such iterative development strategies are common not only for c‐Myc inhibitors but also for other small molecules.

Proteomic and bioinformatic analyses are useful tools for investigating mechanisms of action. Proteomic analysis can reveal the cellular processes that are modulated after drug treatment [69]. In this study, a proteomic approach was applied to identify the activity of cellular processes related to the GO terms “cell death” (GO:0008219) and “cell cycle” (GO:0007049) after ECD treatment in CRC cells. The proteomic analysis results demonstrated that TOP2A was highly enriched in the ECD‐treated group, while RPA1 expression was obviously diminished in this group. The MRN complex participates in the ATM pathway, while RPA1 is a marker of the ATR pathway [70, 71]. Therefore, ECD seems to trigger the DNA damage response (DDR) through the ATM pathway (Fig. 5). This result was confirmed by the quantification of the DNA damage repair marker γ‐H2AX, whose abundance was greatly increased in HT29‐treated cells (Fig. S3). Interestingly, the ATR pathway can be activated downstream of ATM during the G2 phase by the recognition of processed ssDNA ends of DSBs [72]. Moreover, our proteomic analysis revealed an upregulation of CYLD and a downregulation of USP37. The constructed protein–protein interaction network demonstrated that these two proteins might be involved in the regulation of c‐Myc. CYLD is a deubiquitinating enzyme, meaning that it removes specific types of chemical modifiers called ubiquitin moieties from proteins. It functions as a tumor suppressor by negatively regulating several signaling pathways involved in cell proliferation. CYLD has been shown to negatively regulate the expression and activity of c‐Myc in certain cellular contexts through the JNK pathway [73, 74]. USP37 has been shown to stabilize the c‐Myc protein level by removing ubiquitin chains from c‐Myc, thereby preventing its degradation and promoting its oncogenic activity [75].

One of the most common approaches to treat cancer is activation of death mechanisms within cancer cells, [76] and targeting a key cancer driver such as c‐Myc is a highly promising strategy [77]. The longer incubation times significantly increase the likelihood of observing secondary effects. There are several studies of benzoxazine derivatives reported that these derivatives could induce DNA damage by topoisomerase inhibition [78, 79]. In this study, ECD was shown to induce apoptotic cell death, which significantly inhibited PARP activity (Fig. 6A–D). PARP cleavage is associated with reduced DNA repair mechanisms, as the absence of this protein leads to impaired genomic integrity and the accumulation of γ‐H2AX foci (Fig. S3) [80, 81]. Notably, the loss of c‐Myc function due to c‐Myc/MAX interaction by ECD led to its degradation and significantly inhibited essential cell cycle checkpoint markers such as Cyclin B1 and Cyclin D1 (Fig. 6G,H). Therefore, the lack of cell cycle checkpoint activation might result in insufficient DNA repair [82]. Additionally, c‐Myc has been reported to act as a PARP activator [83]. Thus, the inhibition of c‐Myc by ECD could inhibit DNA repair mechanisms through reduced PARP activity. In addition, depletion of c‐Myc has been reported to impair DNA repair mechanisms [84, 85], leading to apoptotic cell death. The antitumorigenic effects of ECD were also confirmed *in vivo* via a CAM assay (Fig. 7E).

The level of c‐Myc was notably decreased in both p53‐wild‐type and p53‐mutant tumor cells (Fig. 7A–D), correlating well with the expression of c‐Myc downstream targets, Cyclin B1 and Cyclin D1 (Fig. 6G,H). The observation that the H‐scores of c‐Myc were not altered in some CAM xenografts might be caused by our therapeutic regime with pretreatment and transplanting surviving cells onto the CAM (Fig. 7H–J). Nevertheless, there was a remarkable decrease in tumor‐forming capability after ECD treatment of cells. The development and evaluation of ECDs should be performed *in ovo* or by i.v. injection and in parallel with other clinically approved drugs.

Overall, our findings lay the groundwork for future research on benzoxazine dimer derivatives for c‐Myc‐targeted therapy. We have identified a promising small molecule that targets c‐Myc by disrupting the c‐Myc/MAX dimer, leading to increased ubiquitin‐mediated proteasomal degradation of the c‐Myc protein. Given that c‐Myc is a key regulator of survival mechanisms, its loss may induce DNA damage due to insufficient DNA repair, resulting in apoptotic cell death. Since c‐Myc is traditionally considered an undruggable protein, these findings highlight ECD as a potential candidate for c‐Myc‐targeted anticancer therapy. Moreover, the DNA damage effect was found after treatment (Fig. S3), so ECD was proposed as a two‐way mechanism compound. Additionally, structural predictions of the binding interaction can provide fundamental insights for developing benzoxazine dimer derivatives and modifying their structures to enhance their affinity and specificity.

## 5 Conclusions

ECD demonstrates the potential as a c‐Myc inhibitor through interference between the c‐Myc/MAX complex, leading to complex disruption. Free‐form c‐Myc undergoes degradation via the ubiquitin proteasomal mechanism. The depletion of c‐Myc protein could impair the DNA damage response, leading to cell cycle arrest and apoptotic cell death. The antitumorigenesis of ECD was exhibited in the CAM model. Therefore, ECD could be a further lead structure for the development of a c‐Myc inhibitor and breakthrough novel cancer treatment.

## Conflict of interest

The authors declare no conflict of interest.

## Author contribution

NS conceived the study, carried out the experiments, analyzed the data, archived the original data, and wrote the first draft of the manuscript. BN and BP performed the computational analysis shown in Fig. 2 and wrote the first draft of the manuscript. WW synthesized and characterized the test compounds, generated Fig. 7, and wrote the first draft of the manuscript. NP and SR conducted proteomic analysis. KEW performed immunohistochemical analysis of tissue slices. PC and RSS conceived and supervised the study and finalized the manuscript. All authors gave final approval of the submitted and published version.

## Supporting information


**Fig. S1.** The active and ubiquitination forms of c‐Myc were evaluated after ECD treatment. (A) HT29 and (B) HCT116 cells were treated with 75 μm EMD or 75 μm ECD for 3 h with or without MG132 (10 μm) pretreatment for 1 h. Total c‐Myc, p‐c‐Myc^Ser62^ and p‐c‐Myc^Thr58^ protein levels were measured by western blot analysis. GAPDH protein expression was evaluated to confirm the equal loading of each protein sample (*n* = 3).

**Fig. S2.** Schematic representation of the CAM procedure. The eggs were incubated in 37 C and 60% humidity with gentle rocked for 7 days. Then, the eggs were pinched a small hole to allow the translocation of the air sac to the top of the eggs. After that eggshell was opened rounder window and the embryonic membrane was peeled off. The window was sealed with adhesive tape. At this day, the unfertilized eggs were eliminated. On Day 9, The transplanted cells were pretreatment with ECD at various concentrations (0–100 μm) for 48 h before subjected for matrix gel pellets at concentration of 1 × 10^6^ cells per pellet. The pellets were put between the blood vessels. The eggs were incubated for 5 days before harvesting the tumors at Day 14.

**Fig. S3.** Induction of DNA damage after ECD treatment in CRC cells *in vitro* and *in vivo*. (A) To reveal the dynamics of DNA damage and the DNA damage response after ECD treatment, γ‐H2AX foci formation was determined by immunocytochemical staining. Actin filaments and nuclei were stained with phalloidin‐555 and DAPI, respectively (*n* = 3). The image was taken at 60× magnifications (scale bar: 200 μm) (B, C) CRC cells were treated with various concentrations of ECD (0–100 μm) for 48 h before being used for western blot analysis of DNA damage markers. GAPDH protein expression was evaluated to confirm the equal loading of each protein sample (*n* = 3). (D) The protein abundance of γ‐H2AX was evaluated by immunohistochemical staining of formalin‐fixed paraffin‐embedded (FFPE) tumor tissues harvested from CAM (*n* = 7). The scanned images of CAM sections were acquired at 30× magnification (overview sections, scale bar: 40 μm). (E, F) The immunohistochemistry score was calculated based on the percentage of stained cells per unit area. The samples were classified according to the staining intensity (0 = negative staining, 1 = weak staining, 2 = moderate staining and 3 = strong staining). The **, *** and ***** symbols on the GAPDH bands indicate the use of the same bands for the normalization of other protein markers. The GAPDH** and GAPDH*** bands in (B) were also used in Figs 5I, S5A, respectively. The GAPDH***** band in (C) was also used in Fig. S5B.

**Fig. S4.** The protein expression of Ki67 and Pan‐CK were evaluated. (A, C) The protein expression of Ki67 which is the proliferation marker, and Pan‐CK which was used to represent the tumor area were evaluated by immunohistochemical staining of formalin‐fixed paraffin‐embedded (FFPE) HT29‐ and HCT116‐tumor tissues harvested from CAM (*n* = 7). The scanned images of CAM sections were acquired at 30× magnification (overview sections, scale bar: 100 μm). (B, D) The data score was presented with mean ± SEMs. Multiple comparisons to identify statistically significant differences among multiple groups were performed with one‐way ANOVA, and individual comparisons were then performed with the Scheffe's *post hoc* test.

**Fig. S5.** The p21 accumulation after ECD treatment was evaluated. CRC cells were treated with various concentrations of ECD (0–100 μm) for 48 h before being used for western blot analysis of cell cycle markers. GAPDH protein expression was evaluated to confirm the equal loading of each protein sample. GAPDH*** bands in (A) were also used in Fig. S3B and the GAPDH***** band in (B) was also used in Fig.S3C (*n =* 3). (C) The localization of p21 was revealed by immunohistochemical staining of FFPE tumor tissues harvested from the CAM. The black arrow indicates cytoplasmic p21, and the red arrow indicates nuclear p21. The scanned images of CAM sections were acquired at 30\u00D7 magnification (overview sections, scale bar: 100 μm). (D, E) The protein expression of nuclear and cytoplasmic p21 was separately revealed by immunohistochemical staining of FFPE tumor tissues harvested from the CAM. The nuclear and cytoplasmic p21 scores of controls and ECD‐treated tumor sections determined by IHC staining were compared. The data are presented as the means ± SEMs (*n* = 7).

**Fig. S6.** ^1^H NMR (Left) and ^13^C NMR (right) profile of each benzoxazine dimer derivatives were demonstrated. (A) ^1^H NMR (Left) and ^13^C NMR (right) profile of 24CD (B) ^1^H NMR (Left) and ^13^C NMR (right) profile of 24MD (C) ^1^H NMR (Left) and ^13^C NMR (right) profile of ECD (D) ^1^H NMR (Left) and ^13^C NMR (right) profile of EMD (E) ^1^H NMR (Left) and ^13^C NMR (right) profile of MCD (F) ^1^H NMR (Left) and ^13^C NMR (right) profile of MMD (G) ^1^H NMR (Left) and ^13^C NMR (right) profile of MeCD (H) ^1^H NMR (Left) and ^13^C NMR (right) profile of MeMD.

**Table S1.** List of proteins related to cell cycle (GO:0007049) in HT29 after treatment of ECD at 75 μm for 24 h.
**Table S2.** list of proteins related to cell death (GO:0008219) in HT29 after treatment of ECD at 75 μm for 24 h.

## Data Availability

The amino acid sequence was obtained from UniProtKB (https://www.uniprot.org/) with accession number P01106. The protein–protein interaction network was analyzed using the STITCH database (http://stitch.embl.de/). Other supplemental data are available online, and the datasets used in this study can be obtained from the corresponding author upon request.
