# Supplementary figures and images for: A synthetic benzoxazine dimer derivative targets c‐Myc to inhibit colorectal cancer progression
Source: Mol Oncol. 2025 Oct 14;20(3):688–708. doi: 10.1002/1878-0261.70127 (PMC13042603; doi:10.1002/1878-0261.70127)

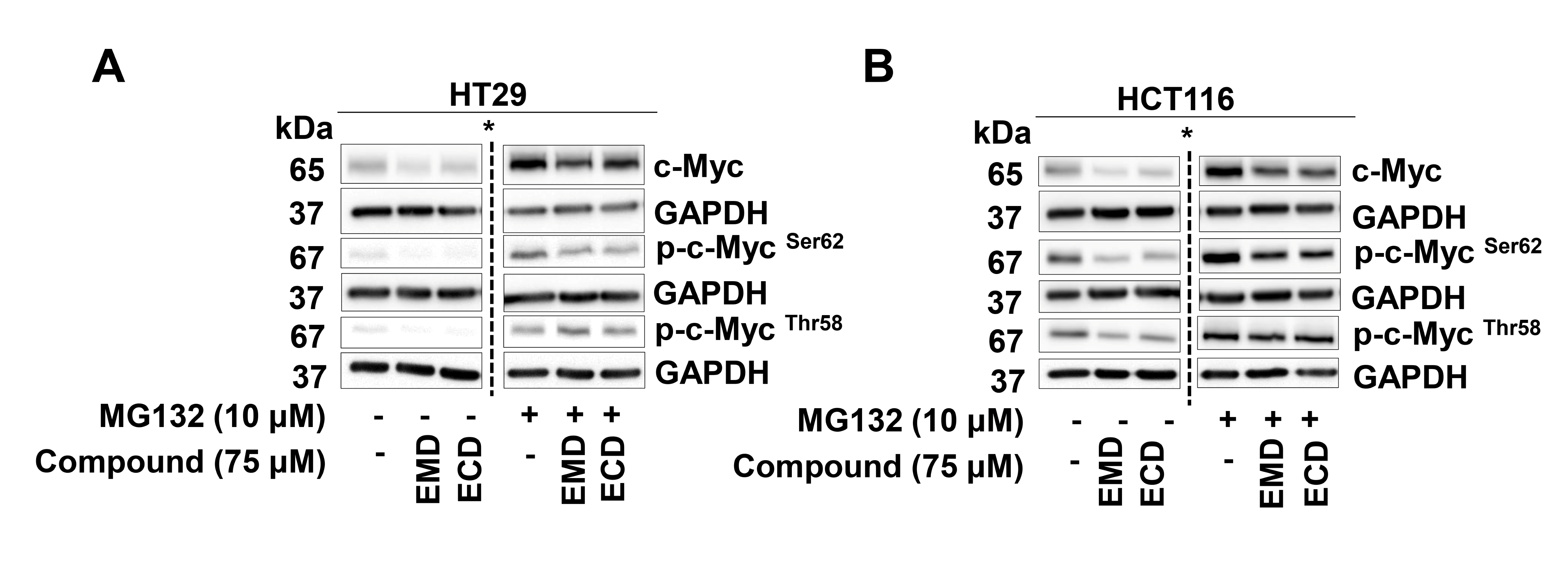

Supplement: Supplementary file 1 — Fig. S1. The active and ubiquitination forms of c‐Myc were evaluated after ECD treatment. (A) HT29 and (B) HCT116 cells were treated with 75 μm EMD or 75 μm ECD for 3 h with or without MG132 (10 μm) pretreatment for 1 h. Total c‐Myc, p‐c‐MycSer62 and p‐c‐MycThr58 protein levels were measured by western blot analysis. GAPDH protein expression was evaluated to confirm the equal loading of each protein sample (n = 3). [file MOL2-20-688-s005.tif]

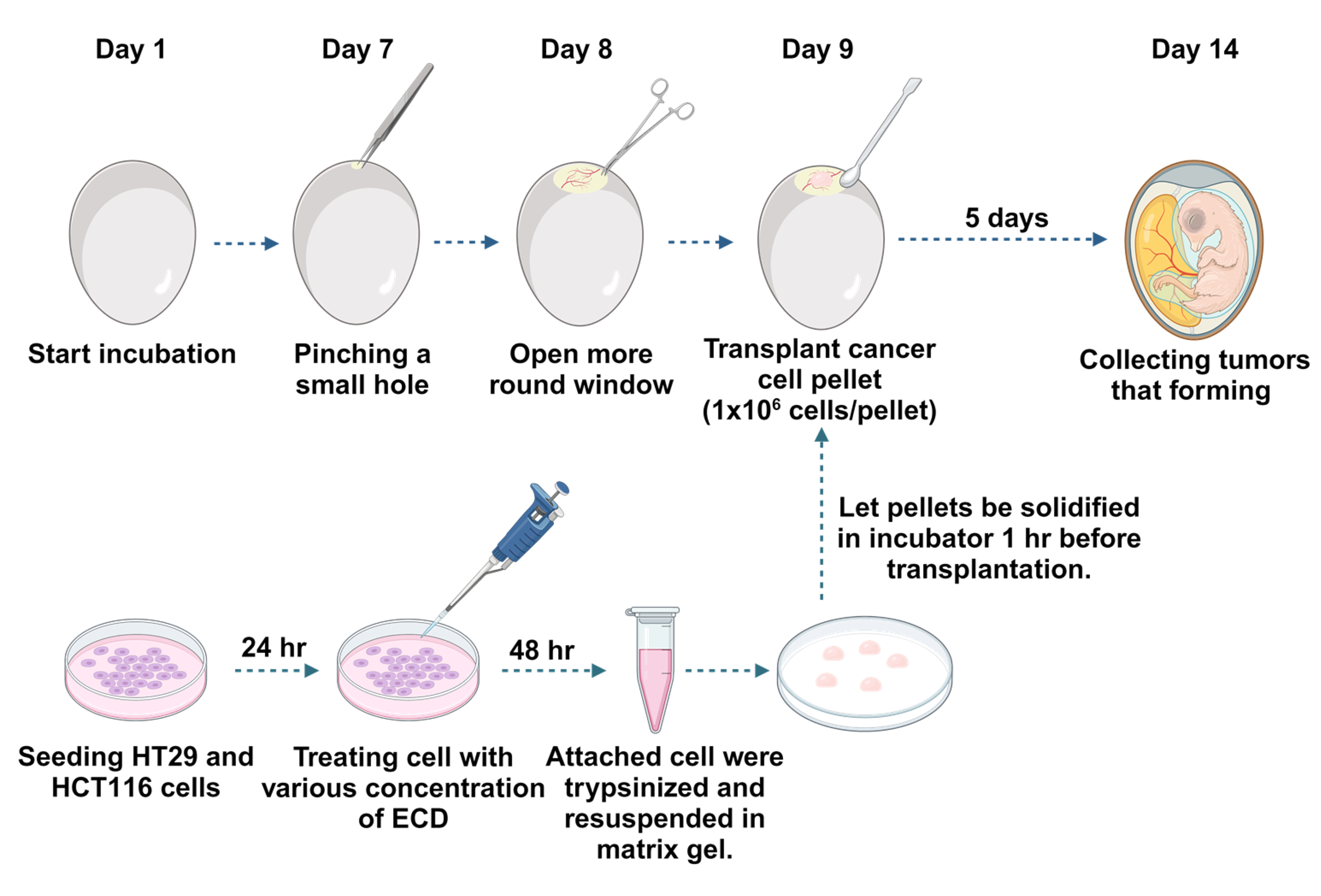

Supplement: Supplementary file 2 — Fig. S2. Schematic representation of the CAM procedure. The eggs were incubated in 37 C and 60% humidity with gentle rocked for 7 days. Then, the eggs were pinched a small hole to allow the translocation of the air sac to the top of the eggs. After that eggshell was opened rounder window and the embryonic membrane was peeled off. The window was sealed with adhesive tape. At this day, the unfertilized eggs were eliminated. On Day 9, The transplanted cells were pretreatment with ECD at various concentrations (0–100 μm) for 48 h before subjected for matrix gel pellets at concentration of 1 × 10^6^ cells per pellet. The pellets were put between the blood vessels. The eggs were incubated for 5 days before harvesting the tumors at Day 14. [file MOL2-20-688-s006.tif]

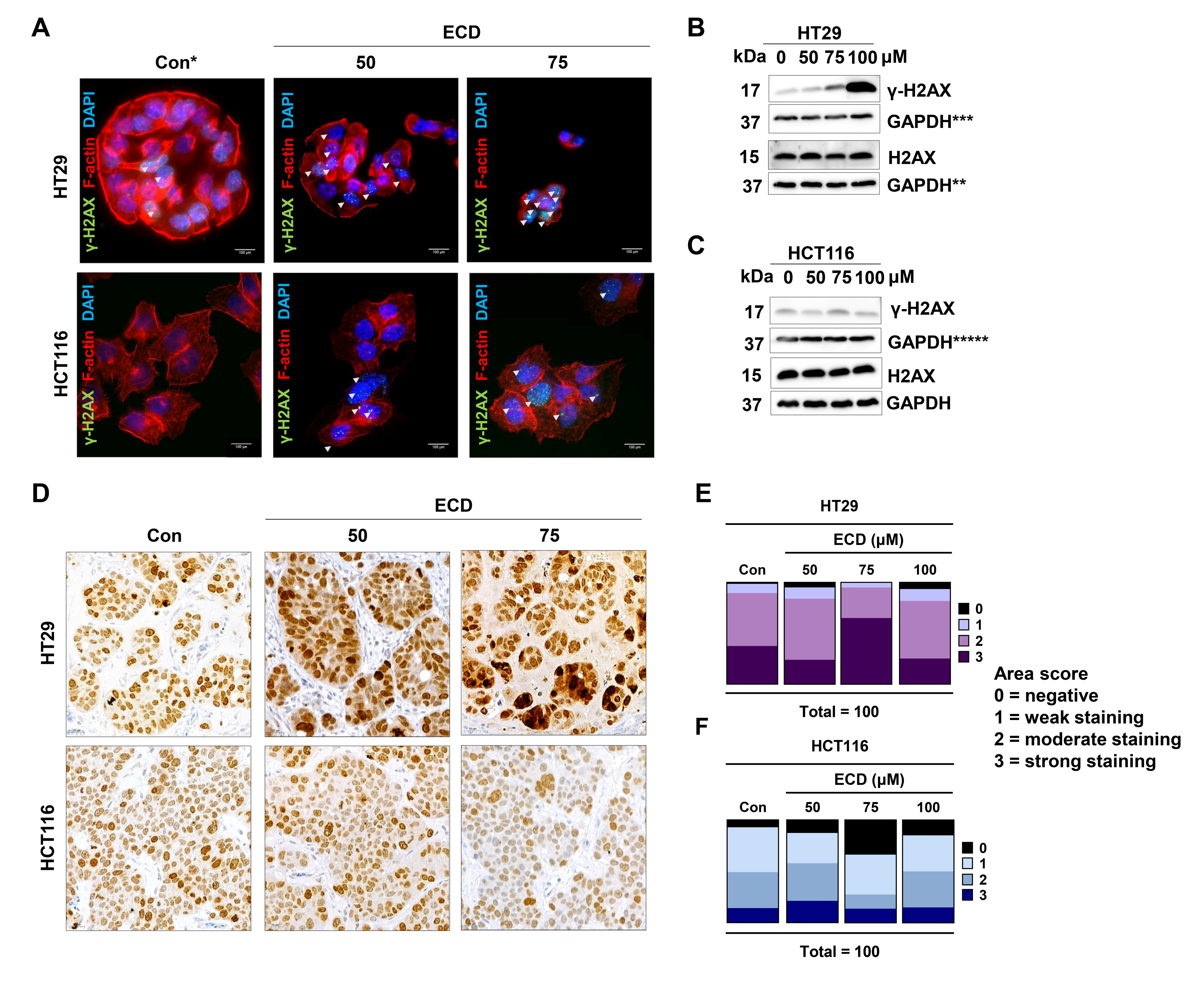

Supplement: Supplementary file 3 — Fig. S3. Induction of DNA damage after ECD treatment in CRC cells in vitro and in vivo. (A) To reveal the dynamics of DNA damage and the DNA damage response after ECD treatment, γ‐H2AX foci formation was determined by immunocytochemical staining. Actin filaments and nuclei were stained with phalloidin‐555 and DAPI, respectively (n = 3). The image was taken at 60× magnifications (scale bar: 200 μm) (B, C) CRC cells were treated with various concentrations of ECD (0–100 μm) for 48 h before being used for western blot analysis of DNA damage markers. GAPDH protein expression was evaluated to confirm the equal loading of each protein sample (n = 3). (D) The protein abundance of γ‐H2AX was evaluated by immunohistochemical staining of formalin‐fixed paraffin‐embedded (FFPE) tumor tissues harvested from CAM (n = 7). The scanned images of CAM sections were acquired at 30× magnification (overview sections, scale bar: 40 μm). (E, F) The immunohistochemistry score was calculated based on the percentage of stained cells per unit area. The samples were classified according to the staining intensity (0 = negative staining, 1 = weak staining, 2 = moderate staining and 3 = strong staining). The **, *** and ***** symbols on the GAPDH bands indicate the use of the same bands for the normalization of other protein markers. The GAPDH** and GAPDH*** bands in (B) were also used in Figs 5I, S5A, respectively. The GAPDH***** band in (C) was also used in Fig. S5B. [file MOL2-20-688-s002.tif]

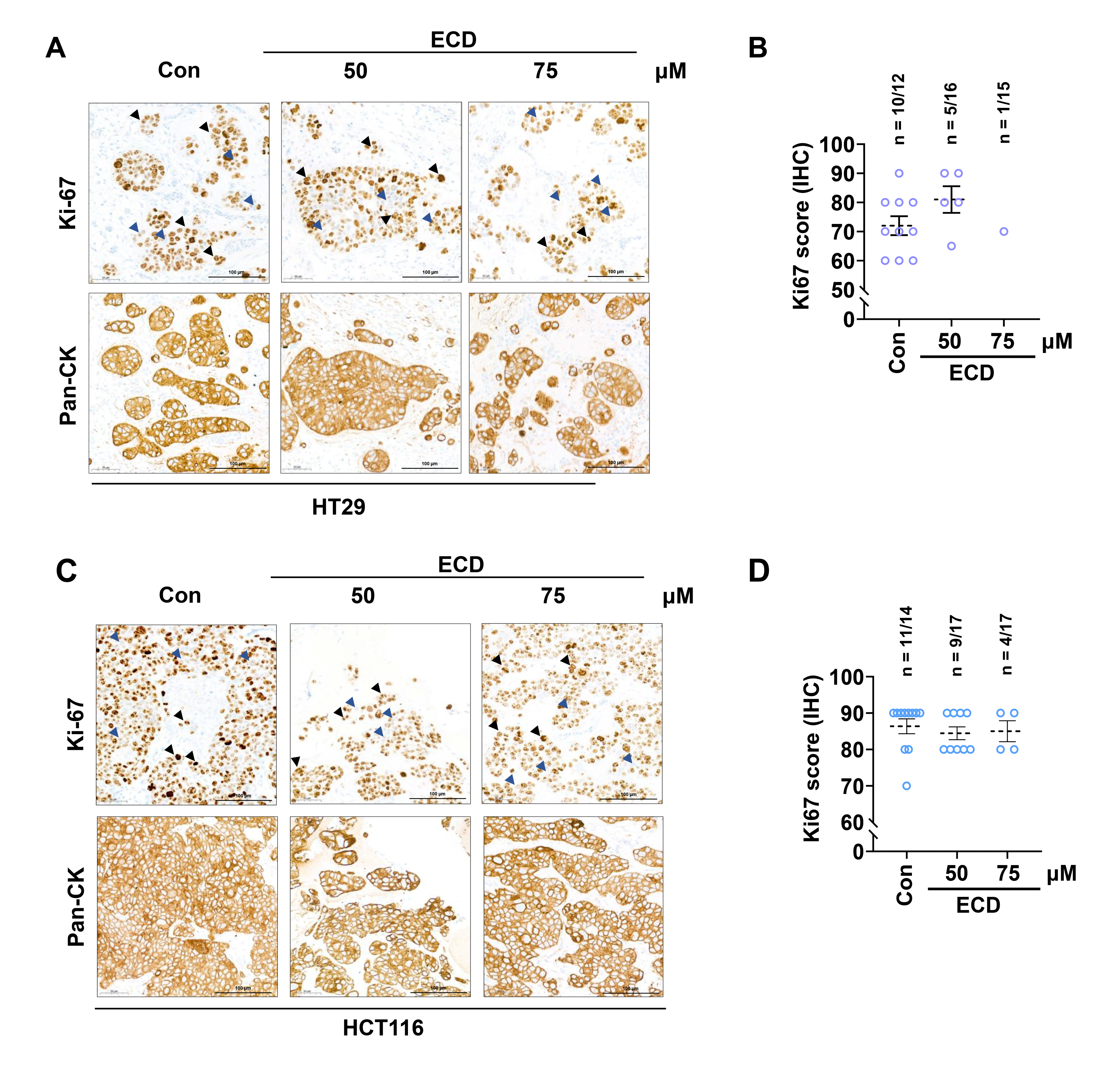

Supplement: Supplementary file 4 — Fig. S4. The protein expression of Ki67 and Pan‐CK were evaluated. (A, C) The protein expression of Ki67 which is the proliferation marker, and Pan‐CK which was used to represent the tumor area were evaluated by immunohistochemical staining of formalin‐fixed paraffin‐embedded (FFPE) HT29‐ and HCT116‐tumor tissues harvested from CAM (n = 7). The scanned images of CAM sections were acquired at 30× magnification (overview sections, scale bar: 100 μm). (B, D) The data score was presented with mean ± SEMs. Multiple comparisons to identify statistically significant differences among multiple groups were performed with one‐way ANOVA, and individual comparisons were then performed with the Scheffe's post hoc test. [file MOL2-20-688-s004.tif]

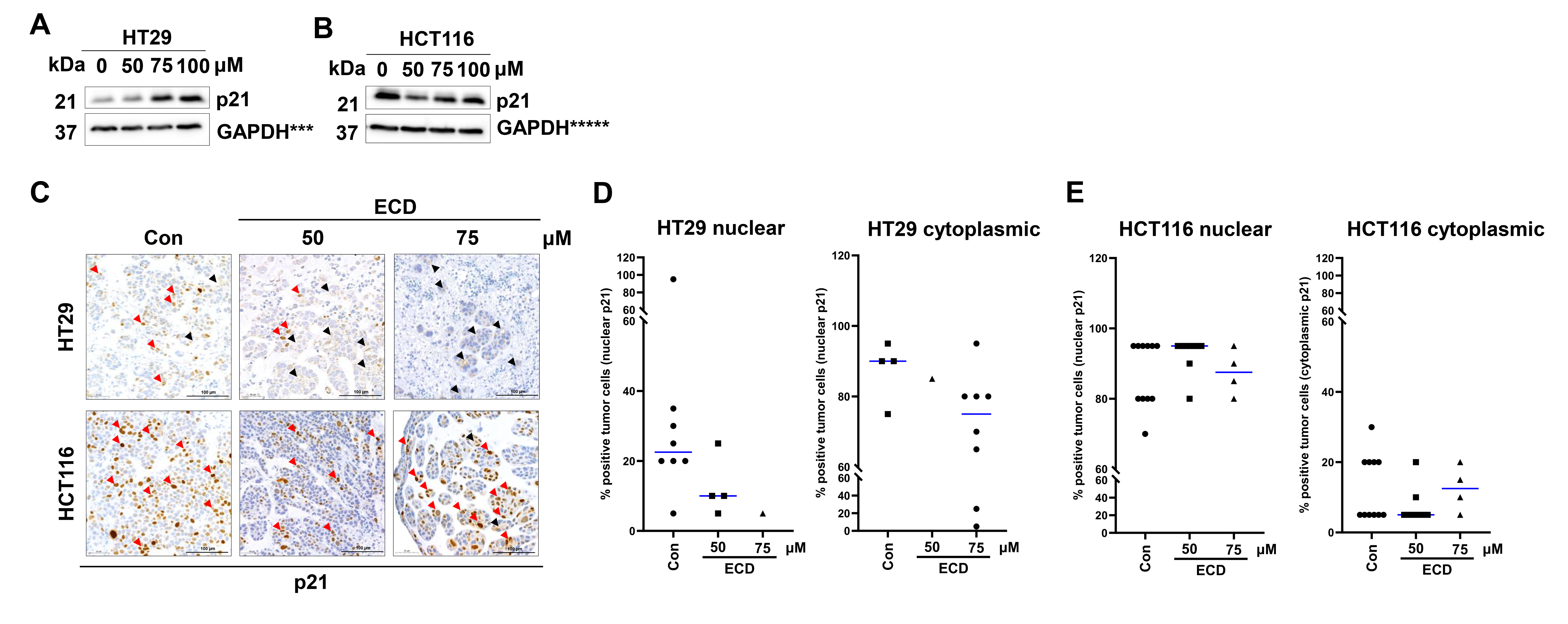

Supplement: Supplementary file 5 — Fig. S5. The p21 accumulation after ECD treatment was evaluated. CRC cells were treated with various concentrations of ECD (0–100 μm) for 48 h before being used for western blot analysis of cell cycle markers. GAPDH protein expression was evaluated to confirm the equal loading of each protein sample. GAPDH*** bands in (A) were also used in Fig. S3B and the GAPDH***** band in (B) was also used in Fig.S3C (n = 3). (C) The localization of p21 was revealed by immunohistochemical staining of FFPE tumor tissues harvested from the CAM. The black arrow indicates cytoplasmic p21, and the red arrow indicates nuclear p21. The scanned images of CAM sections were acquired at 30\u00D7 magnification (overview sections, scale bar: 100 μm). (D, E) The protein expression of nuclear and cytoplasmic p21 was separately revealed by immunohistochemical staining of FFPE tumor tissues harvested from the CAM. The nuclear and cytoplasmic p21 scores of controls and ECD‐treated tumor sections determined by IHC staining were compared. The data are presented as the means ± SEMs (n = 7). [file MOL2-20-688-s001.tif]

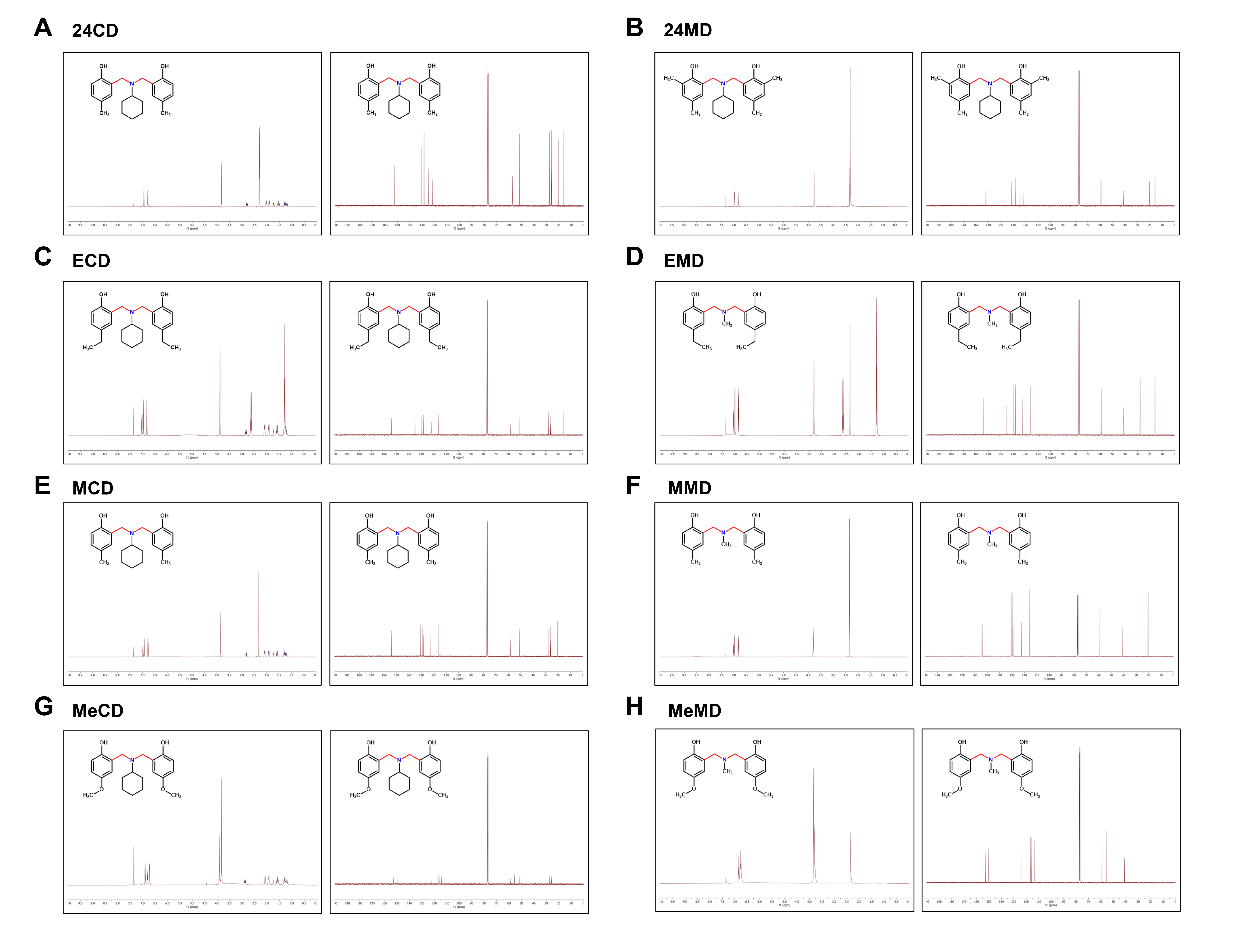

Supplement: Supplementary file 6 — Fig. S6. 1H NMR (Left) and 13C NMR (right) profile of each benzoxazine dimer derivatives were demonstrated. (A) 1H NMR (Left) and 13C NMR (right) profile of 24CD (B) 1H NMR (Left) and 13C NMR (right) profile of 24MD (C) 1H NMR (Left) and 13C NMR (right) profile of ECD (D) 1H NMR (Left) and 13C NMR (right) profile of EMD (E) 1H NMR (Left) and 13C NMR (right) profile of MCD (F) 1H NMR (Left) and 13C NMR (right) profile of MMD (G) 1H NMR (Left) and 13C NMR (right) profile of MeCD (H) 1H NMR (Left) and 13C NMR (right) profile of MeMD. [file MOL2-20-688-s007.tif]
