# Supplementary material for: A synthetic benzoxazine dimer derivative targets c‐Myc to inhibit colorectal cancer progression
Source: Mol Oncol. 2025 Oct 14;20(3):688–708. doi: 10.1002/1878-0261.70127 (PMC13042603; doi:10.1002/1878-0261.70127)
Supplement: Supplementary file 7 — Table S1. List of proteins related to cell cycle (GO:0007049) in HT29 after treatment of ECD at 75 μm for 24 h. Table S2. list of proteins related to cell death (GO:0008219) in HT29 after treatment of ECD at 75 μm for 24 h. [file MOL2-20-688-s003.docx]

**SUPPLEMENTARY TABLES**

**Table S1**.

| Protein name | Gene symbol | ID Score | Log_2_ abundance | |
| --- | --- | --- | --- | --- |
|  |  |  | Con | ECD |
| cell cycle (GO:0007049) | | | | |
| Ras GTPase-activating-like protein IQGAP2 | *IQGAP2* | 9.03 | 12.79462 | 14.14163 |
| Rac GTPase-activating protein 1 | *RACGAP1* | 3.58 | 6.278262 | 8.759899 |
| Ras GTPase-activating-like protein IQGAP3 | *IQGAP3* | 3.53 | 7.523859 | 7.913122 |
| Replication protein A 70 kDa DNA-binding subunit | *RPA1* | 4.86 | 1.779389 | 0 |
| Spindle and kinetochore-associated protein 1 | *SKA1* | 6.61 | 8.955277 | 12.38269 |
| Rotatin | *RTTN* | 9.03 | 8.477213 | 15.25273 |
| Rho-associated protein kinase 2 | *ROCK2* | 10.23 | 18.04752 | 15.04213 |
| Structural maintenance of chromosomes protein 1A | *SMC1A* | 11.39 | 13.42625 | 11.93173 |
| Cyclin-P | *CCNP* | 8.2 | 14.20121 | 12.87902 |
| HAUS augmin-like complex subunit 7 | *HAUS7* | 7.26 | 15.63036 | 13.39005 |
| Anaphase-promoting complex subunit 1 | *ANAPC1* | 3.19 | 10.86888 | 13.08328 |
| Serine_threonine-protein phosphatase 2A activator | *PTPA* | 4.56 | 11.00086 | 11.18455 |
| Coiled-coil domain-containing protein 57 | *CCDC57* | 17.06 | 11.30634 | 11.54764 |
| Centromere protein X | *CENPX* | 5.85 | 9.865584 | 11.77431 |
| Condensin complex subunit 1 | *NCAPD2* | 18.95 | 8.292563 | 3.396569 |
| Cytoplasmic dynein 1 heavy chain 1 | *DYNC1H1* | 6.96 | 9.593497 | 11.02344 |
| WD repeat and HMG-box DNA-binding protein 1 | *WDHD1* | 5.32 | 18.63353 | 15.96624 |
| Centromere protein C | *CENPC* | 5.99 | 7.200229 | 9.886299 |
| SOSS complex subunit B2 | *NABP1* | 20.65 | 9.50128 | 9.544238 |
| Myosin-14 | *MYH14* | 1.84 | 6.950151 | 15.51254 |
| Protein Spire Homolog 1 | *SPIRE1* | 7.89 | 12.08072 | 7.737389 |
| Partitioning defective 6 homolog alpha | *PARD6A* | 3.17 | 13.3166 | 15.68749 |
| DNA topoisomerase 2-binding protein 1 | *TOPBP1* | 4.22 | 1.798218 | 4.57758 |
| Double-strand-break repair protein rad21 homolog | *RAD21* | 2.37 | 7.926827 | 6.535694 |
| DNA polymerase alpha catalytic subunit | *POLA1* | 11.34 | 3.128459 | 12.0315 |
| Nucleolar and spindle-associated protein 1 | *NUSAP1* | 1.99 | 13.35339 | 10.1116 |
| CDK5 regulatory subunit-associated protein 2 | *CDK5RAP2* | 12.24 | 13.41954 | 10.51905 |
| Tubulin gamma-1 chain | *TUBG1* | 7.05 | 7.257333 | 6.945334 |
| Structure-specific endonuclease subunit SLX4 | *SLX4* | 3.63 | 3.010384 | 6.48908 |
| EF-hand domain-containing family member C2 | *EFHC2* | 3.77 | 10.45834 | 7.819796 |
| G1_S-specific cyclin-E2 | *CCNE2* | 4.74 | 10.95676 | 8.62529 |
| DNA topoisomerase 1 | *TOP1* | 2.18 | 11.34501 | 13.32521 |
| Centrosomal protein of 63 kDa | *CEP63* | 1.2 | 5.084184 | 7.122064 |
| Chromosome-associated kinesin KIF4B | *KIF4B* | 2.83 | 3.90125 | 7.713616 |
| Membrane-anchored junction protein | *MAJIN* | 1.58 | 9.381501 | 8.513542 |
| Septin-11 | *SEPTIN11* | 16.77 | 2.668912 | 3.680132 |
| Centrin-1 | *CETN1* | 3.85 | 3.860048 | 11.699 |
| G-protein-signaling modulator 2 | *GPSM2* | 2.83 | 8.290549 | 5.963824 |
| Clathrin heavy chain 1 | *CLTC* | 3.26 | 12.10237 | 12.15138 |
| DNA replication licensing factor MCM3 | *MCM3* | 4.1 | 14.07748 | 16.8186 |
| Centrosomal protein of 120 kDa | *CEP120* | 5.43 | 10.99546 | 10.84164 |
| HAUS augmin-like complex subunit 8 | *HAUS8* | 7.74 | 14.83343 | 8.91959 |
| DNA replication licensing factor MCM2 | *MCM2* | 11.74 | 2.433319 | 7.188057 |
| Synaptonemal complex protein 2 | *SYCP2* | 10.74 | 8.639362 | 5.991693 |
| Meiosis-specific coiled-coil domain-containing protein MEIOC | *MEIOC* | 1.99 | 12.48857 | 11.50632 |
| Spindle assembly abnormal protein 6 homolog | *SASS6* | 4.37 | 9.528301 | 11.13087 |
| HAUS augmin-like complex subunit 3 | *HAUS3* | 3.38 | 11.37375 | 15.07204 |
| Meiosis-specific with OB domain-containing protein | *MEIOB* | 7.4 | 8.335217 | 9.219145 |
| Meiotic nuclear division protein 1 homolog | *MND1* | 6.26 | 13.68226 | 10.04256 |
| Golgin subfamily A member 8C | *GOLGA8CP* | 3.34 | 14.83655 | 16.12014 |
| Protein ECT2 | *ECT2* | 3.09 | 12.32528 | 15.28218 |
| Kinesin-like protein KIF11 | *KIF11* | 3.51 | 4.828554 | 7.77098 |
| Gamma-tubulin complex component 5 | *TUBGCP5* | 9.13 | 10.01088 | 15.28397 |
| Centrin-2 | *CETN2* | 9.65 | 11.63754 | 9.027643 |
| DNA replication complex GINS protein PSF3 | *GINS3* | 5.92 | 5.012581 | 4.648437 |
| Condensin-2 complex subunit D3 | *NCAPD3* | 5.76 | 10.04469 | 11.4368 |
| Transitional endoplasmic reticulum ATPase | *VCP* | 10.5 | 6.466759 | 10.02059 |
| Cohesin subunit SA-1 | *STAG1* | 2.62 | 3.256342 | 6.341232 |
| Centromere protein I | *CENPI* | 14.84 | 10.21437 | 9.324431 |
| CLIP-associating protein 2 | *CLASP2* | 2.12 | 5.781233 | 7.498584 |
| Histone-lysine N-methyltransferase SETMAR | *SETMAR* | 1.82 | 15.83661 | 11.35069 |
| Cytoskeleton-associated protein 5 | *CKAP5* | 14.51 | 8.140972 | 6.208694 |
| Serine_threonine-protein kinase Nek11 | *NEK11* | 3.71 | 8.152504 | 7.288269 |
| Ubiquitin carboxyl-terminal hydrolase 37 | *USP37* | 1.52 | 13.64814 | 10.27212 |
| MutS protein homolog 5 | *MSH5* | 11.8 | 13.96382 | 14.08559 |
| Treslin | *TICRR* | 6.38 | 11.01594 | 12.45308 |
| Golgin subfamily A member 8J | *GOLGA8J* | 2.66 | 8.256194 | 4.63748 |
| Kinesin-like protein KIF18A | *KIF18A* | 1.02 | 11.89447 | 13.91184 |
| Myomegalin | *PDE4DIP* | 10.16 | 9.244387 | 7.956708 |
| Rho-associated protein kinase 1 | *ROCK1* | 10.79 | 8.091252 | 10.00242 |
| Kinetochore protein NDC80 homolog | *NDC80* | 5.97 | 1.823062 | 8.664646 |
| Nipped-B-like protein | *NIPBL* | 4.76 | 6.404332 | 14.50963 |
| WD repeat-containing protein 62 | *WDR62* | 11.17 | 20.15619 | 19.42648 |
| Protein SIX6OS1 | *SIX6OS1* | 7.13 | 5.991041 | 9.895004 |
| Homologous-pairing protein 2 homolog | *PSMC3IP* | 2.85 | 11.71307 | 15.27232 |
| Condensin complex subunit 3 | *NCAPG* | 9.05 | 9.870253 | 8.728138 |
| E3 ubiquitin-protein ligase TRIM36 | *TRIM36* | 6.6 | 14.32095 | 6.752448 |
| Telomere repeats-binding bouquet formation protein 1 | *TERB1* | 7.77 | 13.13137 | 8.65802 |
| Mitotic spindle assembly checkpoint protein MAD2A | *MAD2L1* | 4.4 | 8.095002 | 14.18016 |
| Chromosome-associated kinesin KIF4A | *KIF4A* | 2.93 | 13.12811 | 9.387229 |
| DNA topoisomerase I, mitochondrial | *TOP1MT* | 2.36 | 11.87467 | 13.6153 |
| HAUS augmin-like complex subunit 5 | *HAUS5* | 3.27 | 11.17671 | 8.282554 |
| MRN complex-interacting protein | *MRNIP* | 5.98 | 0 | 6.312758 |
| Cohesin subunit SA-2 | *STAG2* | 3.15 | 5.968641 | 5.435621 |
| Putative ATP-dependent RNA helicase DDX11-like protein 8 | *DDX11L8* | 6.23 | 10.23955 | 13.54606 |
| DNA topoisomerase 2-alpha | *TOP2A* | 8.2 | 5.931648 | 14.21319 |
| Kinesin-like protein KIF19 | *KIF19* | 17.21 | 9.77685 | 9.563439 |
| Myosin-10 | *MYH10* | 11.15 | 8.196881 | 9.821121 |
| Septin-4 | *SEPTIN4* | 11.64 | 13.80077 | 11.22312 |
| Cohesin subunit SA-3 | *STAG3* | 9.8 | 9.395099 | 11.571 |
| DNA repair protein RAD50 | *RAD50* | 2.88 | 11.0876 | 9.461047 |
| Kinetochore scaffold 1 | *KNL1* | 2.25 | 14.3976 | 7.269741 |
| Microtubule-associated protein 9 | *MAP9* | 3.69 | 12.39221 | 11.19851 |
| Serine_threonine-protein kinase haspin | *HASPIN* | 15.84 | 13.5334 | 14.96531 |
| Septin-1 | *SEPTIN1* | 8.33 | 13.50871 | 8.490001 |
| Gamma-tubulin complex component 6 | *TUBGCP6* | 2.89 | 6.985186 | 12.0252 |
| Septin-12 | *SEPTIN12* | 5.69 | 4.779371 | 11.36386 |
| Spindle and kinetochore-associated protein 3 | *SKA3* | 2.5 | 7.510852 | 5.790433 |
| Cyclin-G1 | *CCNG1* | 4.07 | 13.52707 | 12.393 |
| Partitioning defective 6 homolog beta | *PARD6B* | 6.94 | 8.018908 | 9.962286 |
| Nibrin | *NBS1* | 2.34 | 0.000000 | 0.000000 |

**Table S2**

| Protein name | Gene symbol | ID Score | Log_2_ abundance | |
| --- | --- | --- | --- | --- |
|  |  |  | Con | ECD |
| cell death (GO:0008219) | | | | |
| Ubiquitin carboxyl-terminal hydrolase CYLD | *CYLD* | 1.2 | 0 | 9.131849 |
| Gasdermin-D | *GSDMD* | 8.29 | 4.630854 | 9.862153 |
| BCL2_adenovirus E1B 19 kDa protein-interacting protein 3 | *BNIP3* | 4.02 | 7.875867 | 11.74271 |
| BCL2_adenovirus E1B 19 kDa protein-interacting protein 2 | *BNIP2* | 3.56 | 5.728718 | 9.430257 |
| Tumor necrosis factor receptor superfamily member 10A | *TNFRSF10A* | 4.35 | 10.10729 | 13.68071 |
| Apoptosis-inducing factor 1, mitochondrial | *AIFM1* | 8.01 | 7.641236 | 9.126249 |
| Caspase-5 | *CASP5* | 1.12 | 9.449664 | 10.62696 |
| Apoptosis-inducing factor 3 | *AIFM3* | 2.75 | 10.94936 | 11.36796 |
| Caspase-8 | *CASP8* | 5.26 | 7.449875 | 7.22378 |
| Induced myeloid leukemia cell differentiation protein Mcl-1 | *MCL1* | 6.51 | 15.0869 | 14.76682 |
| BCL2_adenovirus E1B 19 kDa protein-interacting protein 3-like | *BNIP3L* | 5.85 | 18.57734 | 18.19105 |
| Apoptosis regulator BAX | *BAX* | 11.58 | 18.81987 | 18.20929 |
| Gasdermin-B | *GSDMB* | 7.22 | 7.447157 | 5.669376 |
| Protein prune homolog 2 | *PRUNE2* | 1.92 | 12.84657 | 10.39698 |
| XK-related protein 8 | *XKR8* | 7.04 | 8.80132 | 6.27505 |
| Baculoviral IAP repeat-containing protein 1 | *NAIP* | 16.01 | 10.32601 | 7.272883 |
| Tumor necrosis factor receptor superfamily member 6 | *FAS* | 7.13 | 15.77524 | 10.89106 |
| Caspase-2 | *CASP2* | 6.92 | 15.99736 | 10.74397 |
